# Characterization of Soft Amyloid Cores in Human Prion-Like Proteins

**DOI:** 10.1038/s41598-017-09714-z

**Published:** 2017-09-21

**Authors:** Cristina Batlle, Natalia Sanchez de Groot, Valentin Iglesias, Susanna Navarro, Salvador Ventura

**Affiliations:** 1 Institut de Biotecnologia i de Biomedicina and Departament de Bioquímica i Biologia Molecular, Universitat Autónoma de Barcelona, Bellaterra, 08193 Spain; 2 Bioinformatics and Genomics Programme, Centre for Genomic Regulation (CRG), The Barcelona Institute for Science and Technology, Dr. Aiguader 88, 08003 Barcelona, Spain; 3 Universitat Pompeu Fabra (UPF), Barcelona, Spain

## Abstract

Prion-like behaviour is attracting much attention due to the growing evidences that amyloid-like self-assembly may reach beyond neurodegeneration and be a conserved functional mechanism. The best characterized functional prions correspond to a subset of yeast proteins involved in translation or transcription. Their conformational promiscuity is encoded in Prion Forming Domains (PFDs), usually long and intrinsically disordered protein segments of low complexity. The compositional bias of these regions seems to be important for the transition between soluble and amyloid-like states. We have proposed that the presence of cryptic soft amyloid cores embedded in yeast PFDs can also be important for their assembly and demonstrated their existence and self-propagating abilities. Here, we used an orthogonal approach in the search of human domains that share yeast PFDs compositional bias and exhibit a predicted nucleating core, identifying 535 prion-like candidates. We selected seven proteins involved in transcriptional or translational regulation and associated to disease to characterize the properties of their amyloid cores. All of them self-assemble spontaneously into amyloid-like structures able to propagate their polymeric state. This provides support for the presence of short sequences able to trigger conformational conversion in prion-like human proteins, potentially regulating their functionality.

## Introduction

A broad range of human pathologies, ranging from neurodegenerative conditions such as Alzheimer’s and Parkinson’s diseases, to non-neuronal disorders such as type II diabetes and cataracts, are associated with protein misfolding and aggregation into amyloid-like structures^1^. The self-assembly of proteins into β-sheet-enriched amyloid conformations appears to obey, in many cases, the so-called amyloid-short-stretch hypothesis, according to which, aggregation is first nucleated by the intermolecular contacts formed by a reduced number of specific short regions in the protein^2^. In pathogenic proteins, these stretches are generally around 5–10 residues in length, with a high aggregation propensity and a predominant hydrophobic character^3^.

Prions are proteins able to adopt multiple structural conformations, from which at least one has self-propagating properties, usually an amyloid state^4^. Prions have been traditionally associated with the onset of mammalian neurophatologies^5^. Nevertheless, there are evidences that prion-like mechanisms are not always deleterious and instead they can be used for beneficial purposes^6^. The best characterized set of functional prions has been found in yeast, where they can behave as epigenetic elements, facilitating adaptation to fluctuating environments^7,8^. The conformational promiscuity of yeast prions is encoded in Prion Forming Domains (PFDs)^9^. PFDs are both sufficient and necessary for prion conversion and usually correspond to long and intrinsically disordered segments of low complexity^9^.

The information on the common features shared by yeast PFDs has fueled the development of algorithms aimed to identify similar prion-like domains (PrLDs) and the proteins that contain them at the proteome level^9–17^. Despite PFDs bear the capacity to shift to an amyloid state, classical amyloid prediction algorithms fail to identify them^12^. This observation led to suggest that PFDs assembly is governed by the low complexity and compositional bias common to these domains^9,12^. Examples of algorithms exploiting these features are DIANA^10^, LPS^11^, PrionScan^13^, PLAAC^16^ and PAPA^12^. Remarkably, these computational approaches predict the existence of PrLD-containing proteins in a wide variety of organisms, from prokaryotes to higher eukaryotes^18–23^. In humans, this sub-proteome is enriched in nucleic acid-binding proteins^18,19^. A fraction of these proteins seem to be involved in the formation of membraneless intracellular compartments, like RNA granules, through PrLDs mediated liquid-liquid phase separation^24,25^. Mutations in these domains have been shown to promote an aberrant transition to an aggregated amyloid-like state, the formation of which might led to the onset of neurodegenerative diseases^26,27^.

We have recently proposed that, in addition to composition, the presence of soft amyloid cores inside PFDs and PrLDs could be important for their assembly^28,29^. We rationalized that these assembly-nucleating regions should be longer than classical amyloid stretches, in such a way that the amyloid potential would be more diffusely distributed; each residue having an average lower potency, but with more residues contributing to the assembling force. This will make their aggregation sensitive to protein concentration and seeding. Thus, in our view, the aggregation of prion-like domains shares mechanistic features with those of classical amyloidogenic proteins. This notion was implemented in pWALTZ, an algorithm that predicts the 21-residues long sequence stretch with the highest average amyloid potential in PrLDs^14,15^. This concept was further validated experimentally by demonstrating the existence of such soft amyloid stretches in the prion domains of four of the best characterized yeast prions^30^, as well as in the predicted PrLD of the Rho termination factor of *Clostridium botulinum* ^31^, which later led to the discovery of the first bacterial prion-like protein^32^.

Here, we study the presence of soft amyloid cores in novel putative human prion-like proteins. For this purpose, we performed a stringent computational analysis of the human proteome in the search of domains that, while fulfilling the compositional bias characteristic of PrLDs, would also exhibit a sequence stretch that can potentially nucleate their self-assembly. From this set, we selected seven nucleic acid-binding proteins associated to disease (DDX5, EYA1, ILF3, MED15, NCOA2, PHC1 and TIA1) to structurally characterize the nature of their putative nucleating cores. The results herein indicate that the PrLDs of all these proteins include 21-residues long stretches able to self-assemble spontaneously into non-toxic, β-sheet enriched, Thioflavin-T positive amyloid-like structures displaying self-seeding activity. Therefore, the present work provides compelling experimental evidence for the existence of specific sequences with the potential to trigger the conformational conversion of PrLDs in human proteins.

## Results

### Identification of PrLD soft amyloid cores in human prion-like proteins

The analysis of the 70940 protein sequences in the human proteome was initially performed with PAPA^12^ and further refined with pWALTZ^14^. Both PAPA and pWALTZ algorithms were trained on top of yeast prions; however, they are based on radically different concepts, a suitable composition of the PrLD and the presence of an embedded soft amyloid core, respectively. Sequences identified by these two orthogonal approaches are expected to recapitulate the conformational properties of yeast prions. A total of 663 human proteins were identified by PAPA and later shortlisted using pWALTZ to render a total of 535 polypeptides, encoded by 336 different genes (Table S1).

We wanted to test whether the soft amyloid cores predicted inside the PrLDs of these putative human prion-like proteins could spontaneously self-assemble into amyloid-like conformations and propagate their aggregated state, as we observed before for yeast prions^30^ and the prion-like *C. botulinum* Rho factor^31^. We focused on nucleic acid-binding proteins, both because this molecular function is enriched in our dataset and because most of the experimentally validated yeast prions act in translational or transcriptional regulation. We selected six proteins associated to disease whose prionogenic properties have not been reported before: DDX5, EYA1, ILF3, MED15, NCOA2 and PHC1. We also included in the analysis TIA1, an RNA-binding protein identified by the orthogonal approach for which a prion-like behavior has been already suggested^33^ (Tables 1 and 2).

**Table 1:** Function and implication in disease of the selected human PrLD-containing proteins. Uniprot^77^ and Malacards Human Disease database^78^ were used to determine the function and disease-association for each human PrLD-containing candidate, respectively. All of them are involved in transcriptional o translational regulatory functions and have been associated to common diseases such as cancer or degenerative disorders.

PROTEIN	FUNCTION	DISEASE
DDX5	RNA helicase protein involved in transcriptional regulation	Prostate, breast and colon cancer, leukemia, hepatitis and others.
EYA1	Transcriptional coactivator and protein phosphatase	Branchio-oto-renal syndrome, breast cancer, cataract and others.
ILF3	Facilitates gene expression regulation from transcription to degradation	Cancer, myocardial infarction, chronic kidney disease and others.
MED15	Component of the mediator complex involved in the transcription of RNA-pol II dependent genes	Epicondylitis, prostate cancer, prostatitis and others.
NCOA2	Nuclear receptors and steroid receptors coactivator	Prostate cancer, mesenchymal chondrosarcoma and others.
PHC1	Component of a Polycomb group involved in the maintenance of the transcriptionally repressive state of HOX genes	Microcephaly.
TIA1	Regulates alternative splicing, associated with apoptosis and 3’UTR mRNA binding protein	Amyotrophic Lateral Sclerosis, Hodgkin’s Lymphoma, myopathy, Spinal Muscular Atrophy and others.

**Table 2:** Selected human PrLD amyloid cores. For each PrLD-containing protein it is shown its Uniprot ID, its 21 residues-long amyloid core, the pWALTZ score for this protein region and its Q/N/S/G content.

PROTEIN	UNIPROT ID	PrLD AMYLOID CORE	pWALTZ SCORE	Q/N/S/G (%)
DDX5	P17844	530-TQNGVYSAANYTNGSFGSNFV-550	71.62	52.38
EYA1	Q99502	184-MQGSSFTTSSGIYTGNNSLTN-204	74.69	57.14
ILF3	Q12906	672-YGSYGYGGNSATAGYSQFYSN-692	71.01	57.14
MED15	Q96RN5	184-QQQQQFQAQQSAMQQQFQAVV-204	70.58	61.90
NCOA2	Q15596	1374-HFGQQANTSMYSNNMNINVSM-1394	70.39	52.38
PHC1	P78364	382-QQQQIHLQQKQVVIQQQIAIH-402	74.58	47.62
TIA1	P31483	331-AYGMYGQAWNQQGFNQTQSSA-351	67.81	57.14

**Figure 1:**
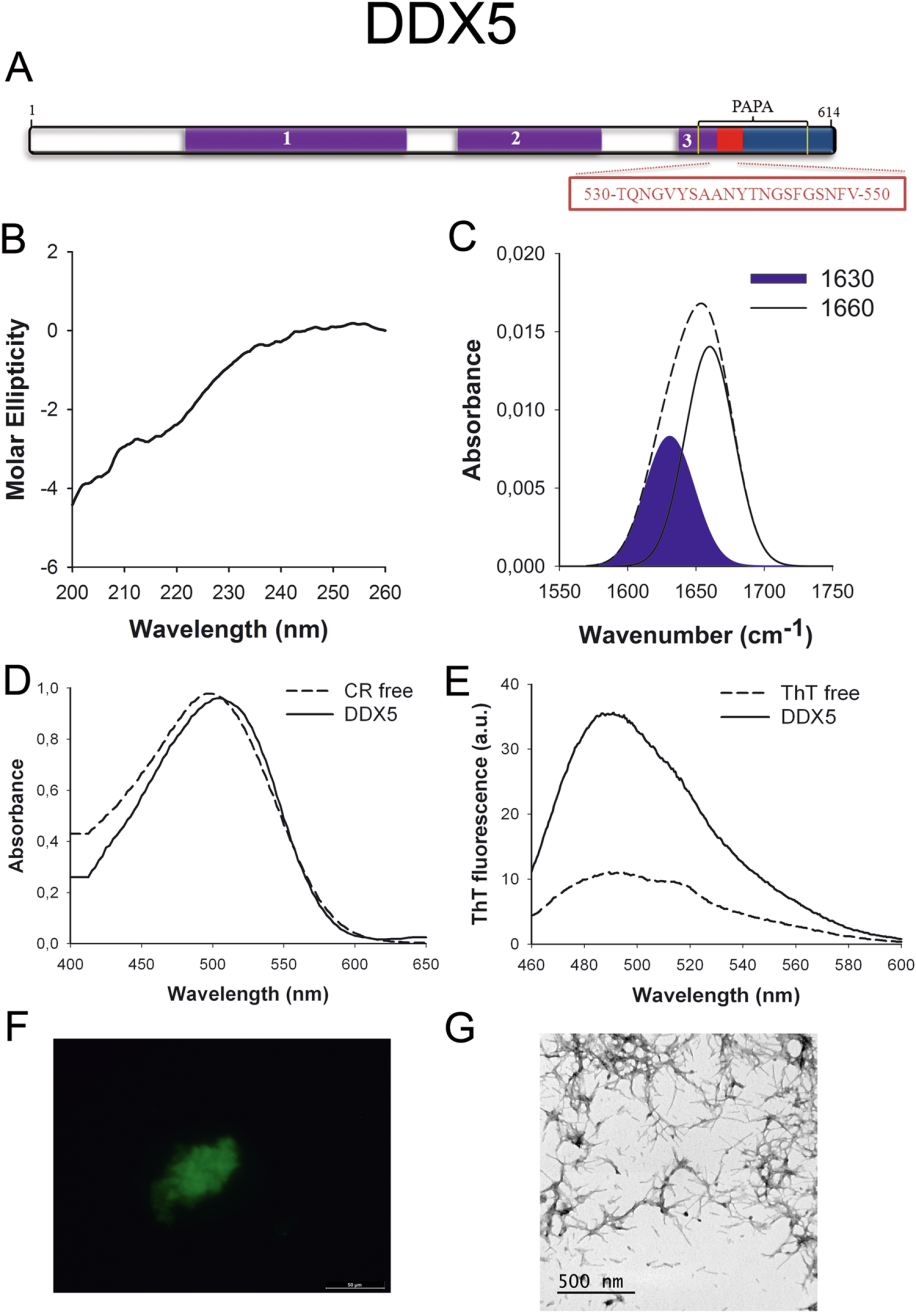
DDX5 PrLD amyloid core. (**A**) DDX5 diagram showing the location of the identified Pfam domains (purple), the amyloid core (red) and the PrLD as predicted by PLAAC (blue) and PAPA (yellow lines). 1 = DEAD domain. 2 = Helicase C domain. 3 = p68-like RNA helicase domain. The sequence of the amyloid core is shown in the box. (**B**) CD spectrum in the far-UV region of 100 μM DDX5 peptide in 5 mM potassium phosphate buffer pH 7.4 before incubation. (**C**) DDX5 peptide FT-IR absorbance spectrum in the amide I region. The dashed line corresponds to the original spectrum, the blue area indicates the contribution of the inter-molecular β-sheet signal to the total area upon Gaussian deconvolution. (**D**) CR absorbance spectrum in the absence (dashed line) and in the presence (solid line) of DDX5 peptide. (**E**) Fluorescence emission spectrum of Th-T in the absence (dashed line) and in the presence (solid line) of DDX5 peptide. (**F**) DDX5 peptide stained with Th-S and observed at 40X magnification using fluorescence microscopy. (**G**) DDX5 peptide representative transmission electron micrograph. The data in panels C to G were collected upon incubation of DDX5 peptide for 2 days in 5 mM potassium phosphate buffer pH 7.4 at 37 °C.

DDX5 (p68) (Fig. 1A) is a member of the DEAD box family of RNA helicases involved in transcriptional regulation^34^ and it is overexpressed in various types of cancers such as those of prostate, breast and colon; promoting cell proliferation and metastasis^35,36^. The C-terminal region containing the predicted PrLD seems to play a role in the interaction of the protein with other components of the transcriptional machinery^37^.

**Figure 2:**
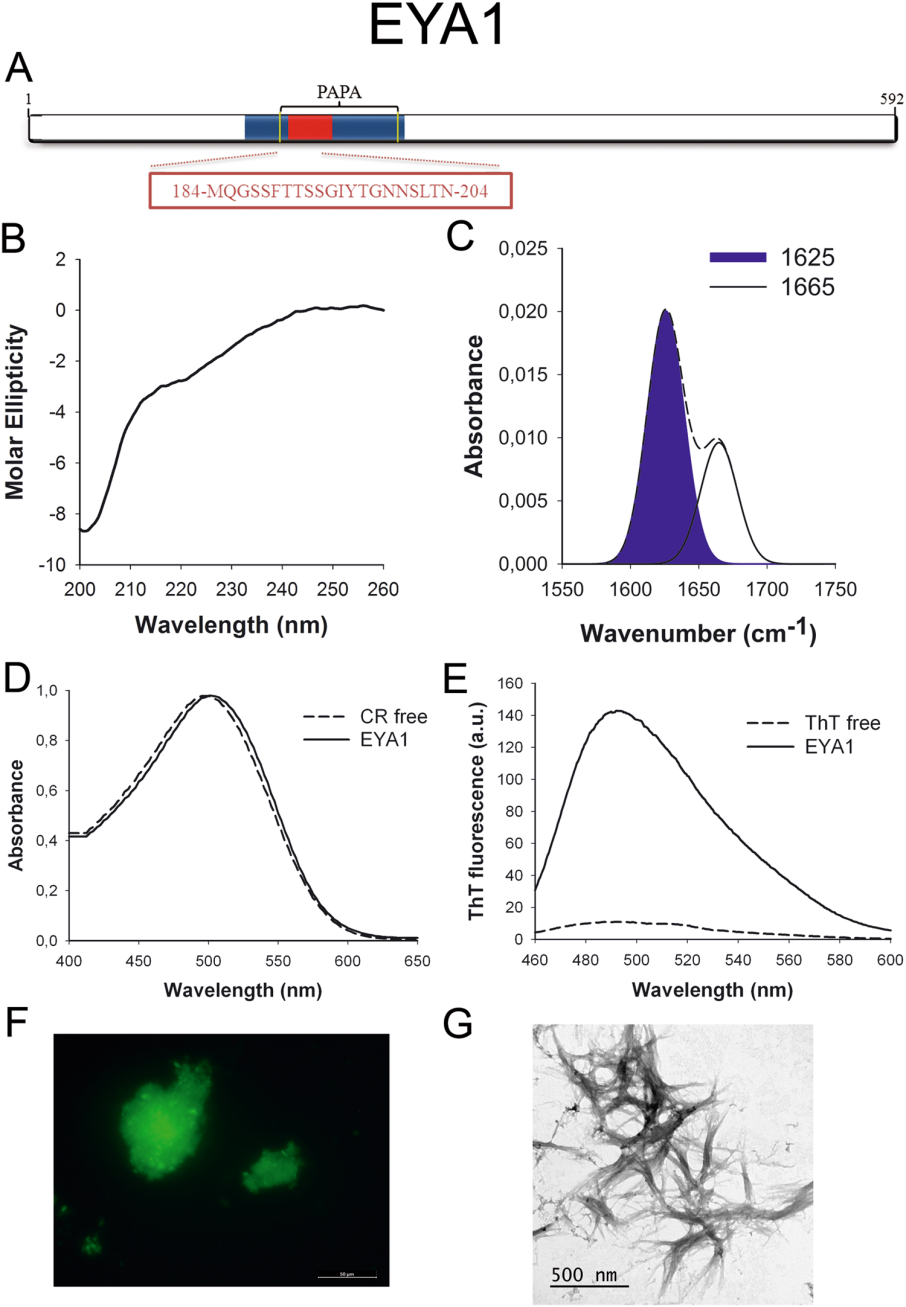
EYA1 PrLD amyloid core. (**A**) EYA1 diagram showing the location of the amyloid core (red) and the PrLD as predicted by PLAAC (blue) and PAPA (yellow lines). The sequence of the amyloid core is shown in the box. (**B**) CD spectrum in the far-UV region of 100 μM EYA1 peptide in 5 mM potassium phosphate buffer pH 7.4 before incubation. (**C**) EYA1 peptide FT-IR absorbance spectrum in the amide I region. The dashed line corresponds to the original spectrum, the blue area indicates the contribution of the inter-molecular β-sheet signal to the total area upon Gaussian deconvolution. (**D**) CR absorbance spectrum in the absence (dashed line) and in the presence (solid line) of EYA1 peptide. (**E**) Fluorescence emission spectrum of Th-T in the absence (dashed line) and in the presence (solid line) of EYA1 peptide. (**F**) EYA1 peptide stained with Th-S and observed at 40X magnification using fluorescence microscopy. (**G**) EYA1 peptide representative transmission electron micrograph. The data in panels C to G were collected upon incubation of EYA1 peptide for 2 days in 5 mM potassium phosphate buffer pH 7.4 at 37 °C.

EYA1 (Fig. 2A) is a transcriptional coactivator and a protein phosphatase with regulatory roles in nephrogenesis^38^. The protein is overexpressed in breast cancer^39^ and mutations in EYA1 gene are associated with branchio-oto-renal syndrome^40^. The N-terminal region containing the PrLD has been reported to function as a transactivation domain^41^.

**Figure 3:**
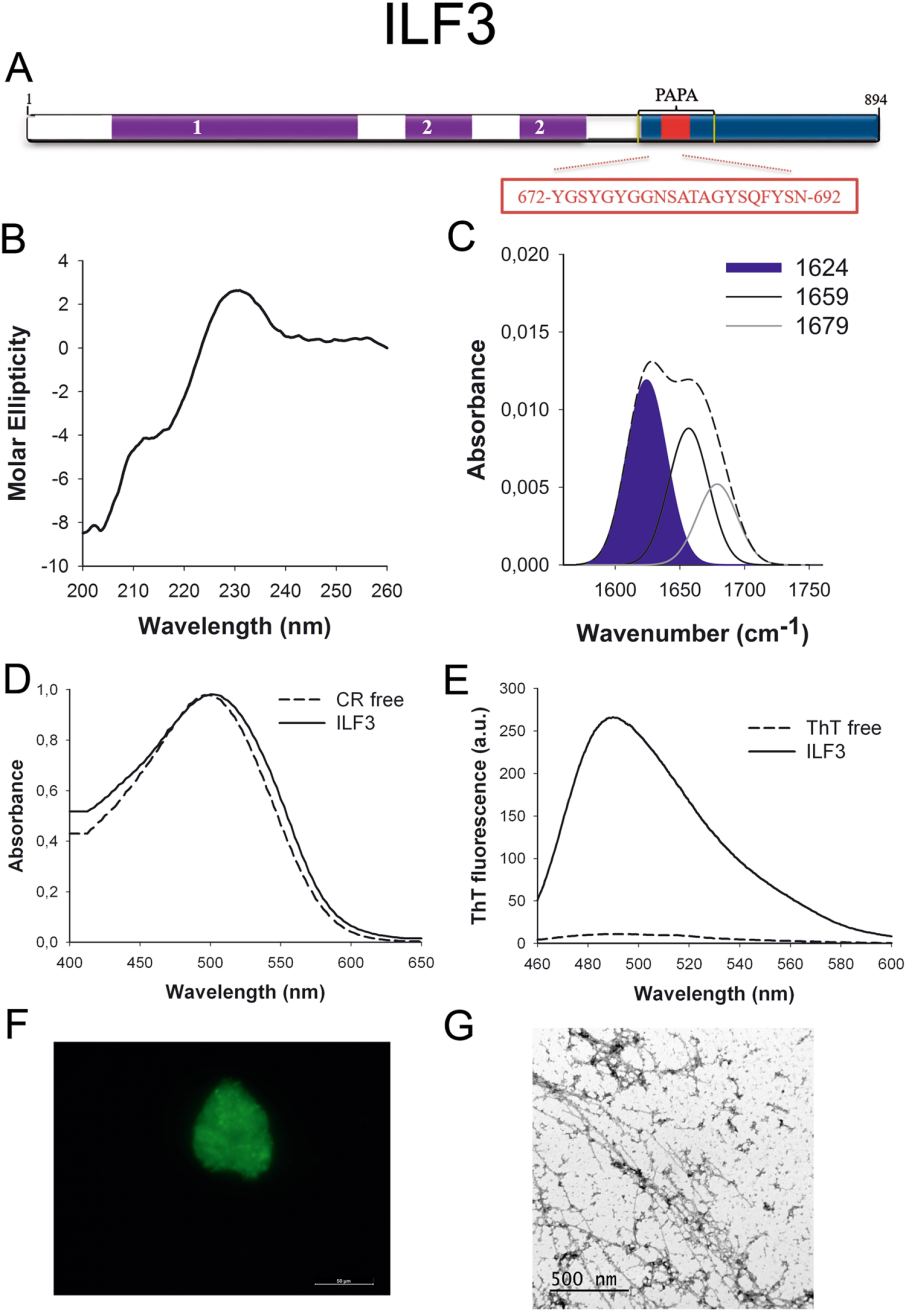
ILF3 PrLD amyloid core. (**A**) ILF3 diagram showing the location of the identified Pfam domains (purple), the amyloid core (red) and the PrLD as predicted by PLAAC (blue) and PAPA (yellow lines). 1 = DZF domain. 2 = dsRNA binding motif. The sequence of the amyloid core is shown in the box. (**B**) CD spectrum in the far-UV region of 100 μM ILF3 peptide in 5 mM potassium phosphate buffer pH 7.4 before incubation. (**C**) ILF3 peptide FT-IR absorbance spectrum in the amide I region. The dashed line corresponds to the original spectrum, the blue area indicates the contribution of the inter-molecular β-sheet signal to the total area upon Gaussian deconvolution. (**D**) CR absorbance spectrum in the absence (dashed line) and in the presence (solid line) of ILF3 peptide. (**E**) Fluorescence emission spectrum of Th-T in the absence (dashed line) and in the presence (solid line) of ILF3 peptide. (**F**) ILF3 peptide stained with Th-S and observed at 40X magnification using fluorescence microscopy. (**G**) ILF3 peptide representative transmission electron micrograph. The data in panels C to G were collected upon incubation of ILF3 peptide for 2 days in 5 mM potassium phosphate buffer pH 7.4 at 37 °C.

ILF3 (Fig. 3A) works in RNA metabolism, from transcription to degradation and it appears to be essential for cellular development^42^. Interestingly, ILF3 participates in ribonucleoprotein (RNP) granules assembly^43^ and it interacts with FUS, a well-characterized prion-like protein^44^.

**Figure 4:**
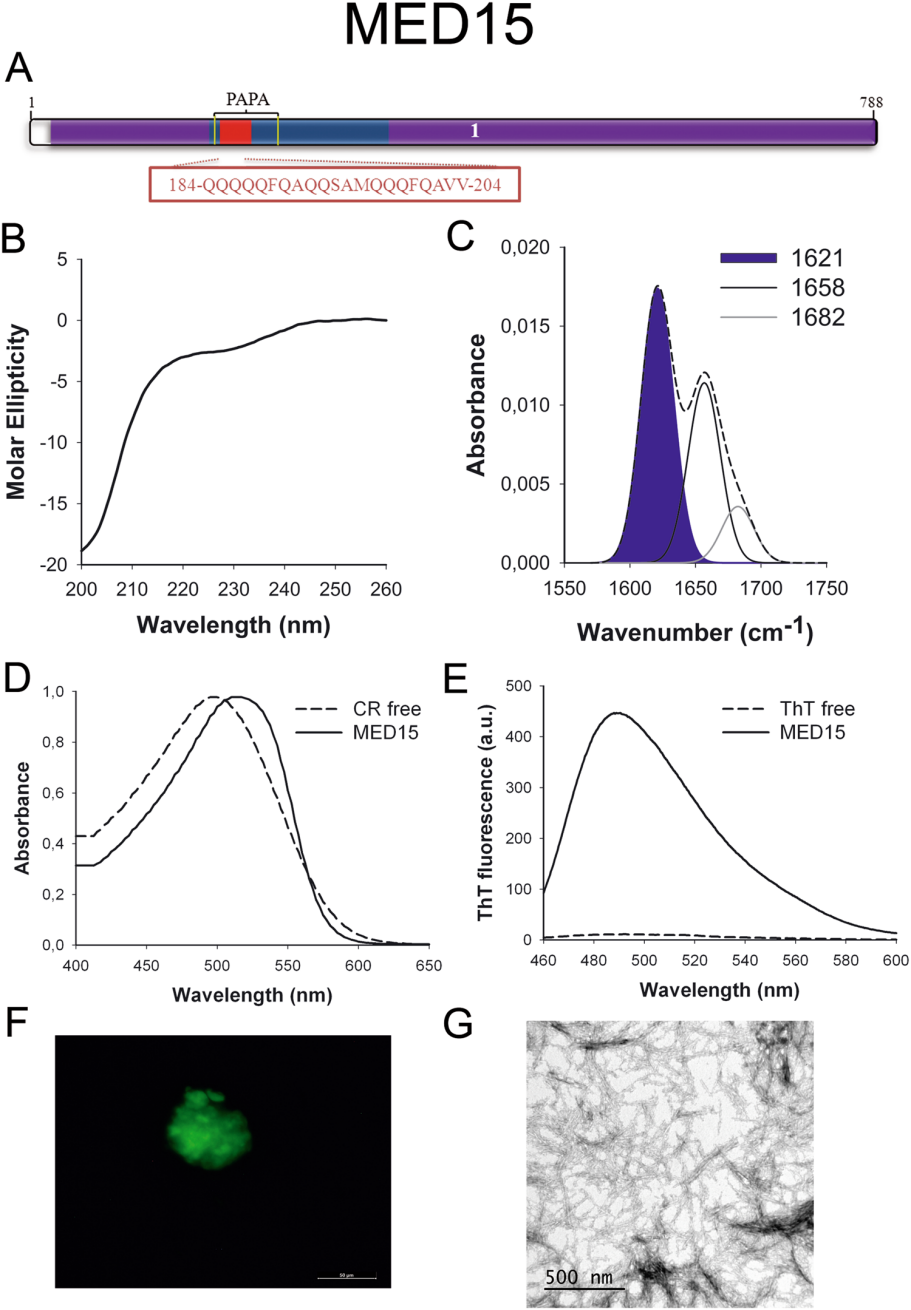
MED15 PrLD amyloid core. (**A**) MED15 diagram showing the location of the identified Pfam domains (purple), the amyloid core (red) and the PrLD as predicted by PLAAC (blue) and PAPA (yellow lines). 1 = MED15 domain. The sequence of the amyloid core is shown in the box. (**B**) CD spectrum in the far-UV region of 100 μM MED15 peptide in 5 mM potassium phosphate buffer pH 7.4 before incubation. (**C**) MED15 peptide FT-IR absorbance spectrum in the amide I region. The dashed line corresponds to the original spectrum, the blue area indicates the contribution of the inter-molecular β-sheet signal to the total area upon Gaussian deconvolution. (**D**) CR absorbance spectrum in the absence (dashed line) and in the presence (solid line) of MED15 peptide. (**E**) Fluorescence emission spectrum of Th-T in the absence (dashed line) and in the presence (solid line) of MED15 peptide. (**F**) MED15 peptide stained with Th-S and observed at 40X magnification using fluorescence microscopy. (**G**) MED15 peptide representative transmission electron micrograph. The data in panels C to G were collected upon incubation of MED15 peptide for 2 days in 5 mM potassium phosphate buffer pH 7.4 at 37 °C.

MED15 (Fig. 4A) is one part of the Mediator complex involved in the transcription of RNA-polymerase II dependent genes^45^. The identified PrLD corresponds to a Q-rich region similar to those accounting for conformational conversion in yeast prions. Indeed, its yeast homolog has been already classified as a prion^46^ with the ability to form amyloid-like structures *in vivo* under stress conditions^47^.

**Figure 5:**
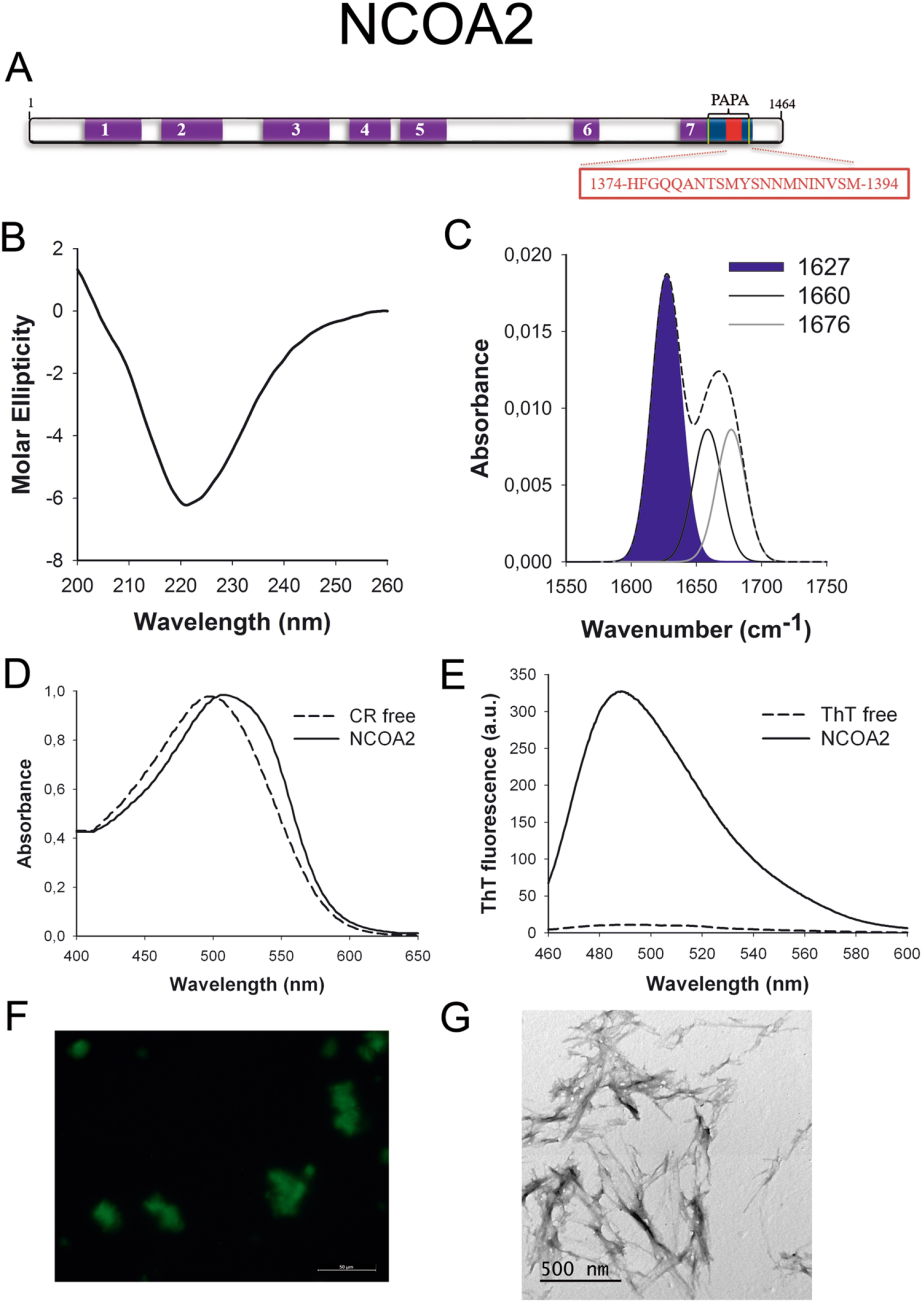
NCOA2 PrLD amyloid core. (**A**) NCOA2 diagram showing the location of the identified Pfam domains (purple), the amyloid core (red) and the PrLD as predicted by PLAAC (blue) and PAPA (yellow lines). 1 = PAS domain. 2 = PAS11 domain. 3 = NCOA_u2 domain. 4 = SRC1 domain. 5 = Duf4927 domain. 6 = Nuc rec co-act domain. 7 = DUF1518. The sequence of the amyloid core is shown in the box. (**B**) CD spectrum in the far-UV region of 100 μM NCOA2 peptide in 5 mM potassium phosphate buffer pH 7.4 before incubation. (**C**) NCOA2 peptide FT-IR absorbance spectrum in the amide I region. The dashed line corresponds to the original spectrum, the blue area indicates the contribution of the inter-molecular β-sheet signal to the total area upon Gaussian deconvolution. (**D**) CR absorbance spectrum in the absence (dashed line) and in the presence (solid line) of NCOA2 peptide. (**E**) Fluorescence emission spectrum of Th-T in the absence (dashed line) and in the presence (solid line) of NCOA2 peptide. (**F**) NCOA2 peptide stained with Th-S and observed at 40X magnification using fluorescence microscopy. (**G**) NCOA2 peptide representative transmission electron micrograph. The data in panels C to G were collected upon incubation of NCOA2 peptide for 2 days in 5 mM potassium phosphate buffer pH 7.4 at 37 °C.

NCOA2 (Fig. 5A) is a transcriptional coactivator for steroid receptors and nuclear receptors acting in the upregulation of DNA expression^48^. It has a tissue-specific role in tumorigenesis, acting as an oncogene in prostate cancer^49^ and as a tumor suppressor in liver cancer^50^. Moreover, NCOA2 is a key player in glucose homeostasis, being involved in Mediator recruitment for glucokinase expression^51^.

**Figure 6:**
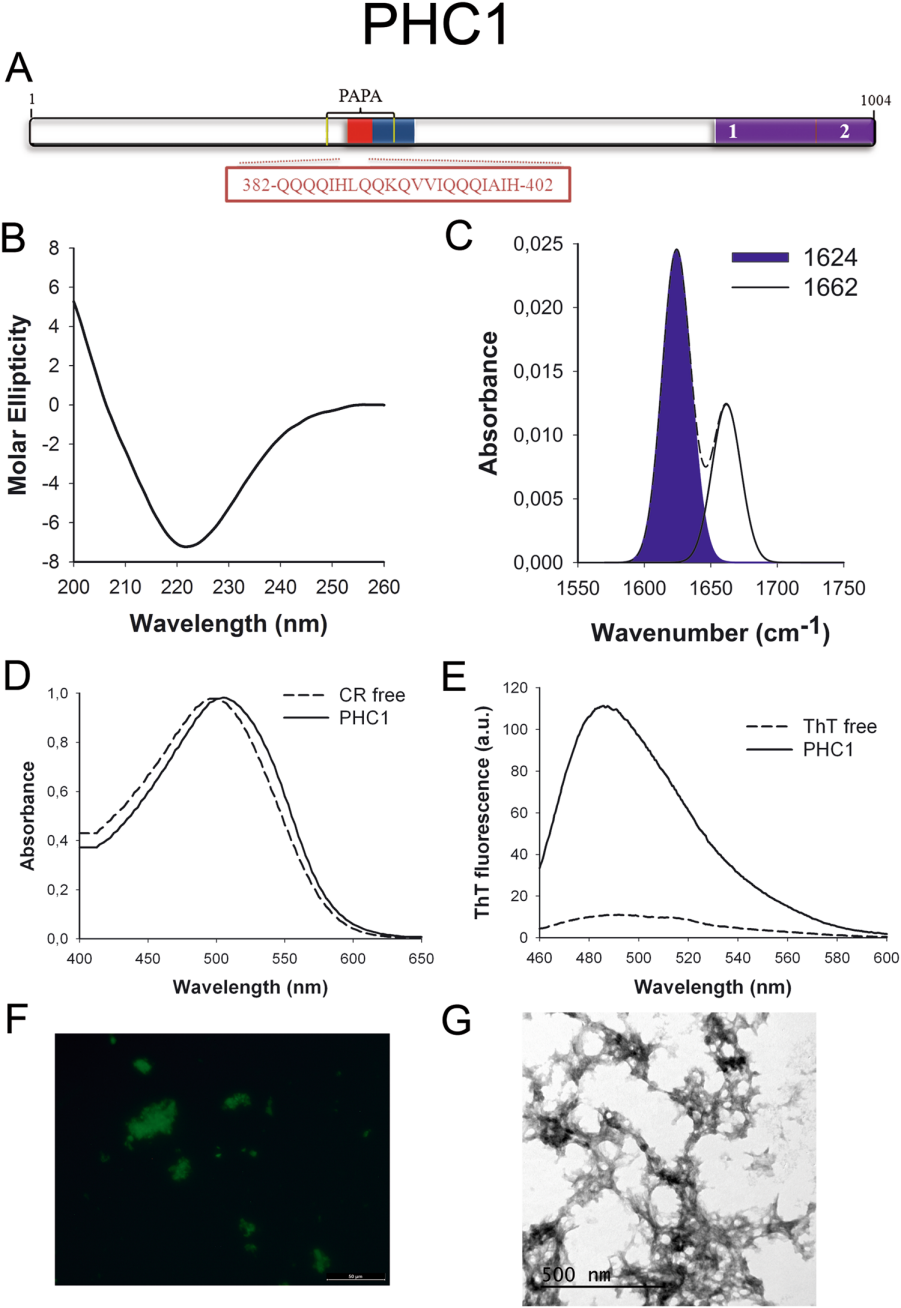
PHC1 PrLD amyloid core. (**A**) PHC1 diagram showing the location of the identified Pfam domains (purple), the amyloid core (red) and the PrLD as predicted by PLAAC (blue) and PAPA (yellow lines). 1 = PHC2 SAM assoc domain. 2 = SAM1 domain. The sequence of the amyloid core is shown in the box. (**B**) CD spectrum in the far-UV region of 100 μM PHC1 peptide in 5 mM potassium phosphate buffer pH 7.4 before incubation. (**C**) PHC1 peptide FT-IR absorbance spectrum in the amide I region. The dashed line corresponds to the original spectrum, the blue area indicates the contribution of the inter-molecular β-sheet signal to the total area upon Gaussian deconvolution. (**D**) CR absorbance spectrum in the absence (dashed line) and in the presence (solid line) of PHC1 peptide. (**E**) Fluorescence emission spectrum of Th-T in the absence (dashed line) and in the presence (solid line) of PHC1 peptide. (**F**) PHC1 peptide stained with Th-S and observed at 40X magnification using fluorescence microscopy. (**G**) PHC1 peptide representative transmission electron micrograph. The data in panels C to G were collected upon incubation of PHC1 peptide for 2 days in 5 mM potassium phosphate buffer pH 7.4 at 37 °C.

PHC1 (Fig. 6A) is one component of the Polycomb complex responsible for cellular differentiation during development. This complex is constituted by gene silencing proteins that repress important developmental regulator genes, including homeotic (HOX) genes^52^. PHC1 is associated with primary microcephaly^53^.

**Figure 7:**
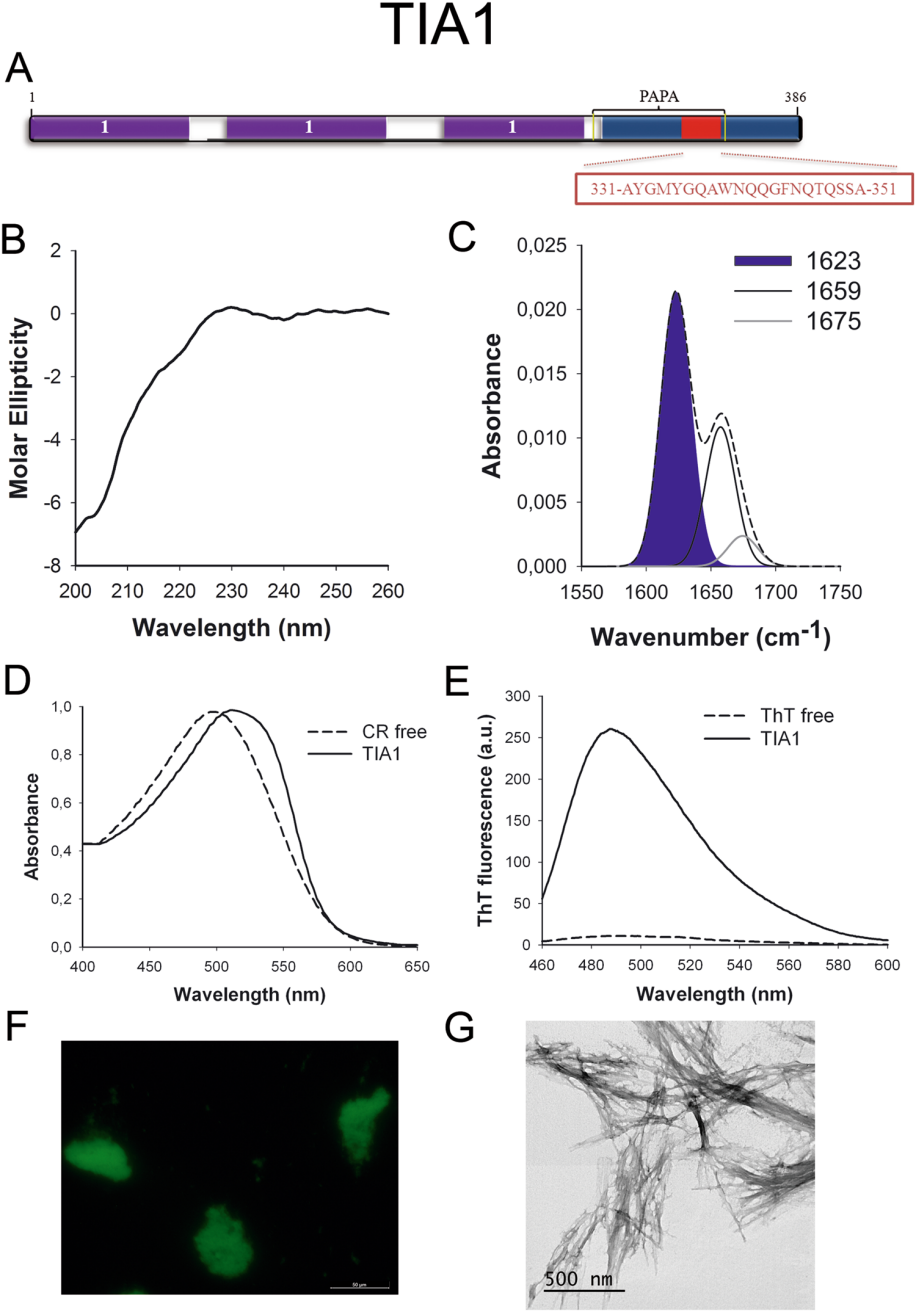
TIA1 PrLD amyloid core. (**A**) TIA1 diagram showing the location of the identified Pfam domains (purple), the amyloid core (red) and the PrLD as predicted by PLAAC (blue) and PAPA (yellow lines). 1 = RNA recognition motif (RRM). The sequence of the amyloid core is shown in the box. (**B**) CD spectrum in the far-UV region of 100 μM TIA1 peptide in 5 mM potassium phosphate buffer pH 7.4 before incubation. (**C**) TIA1 peptide FT-IR absorbance spectrum in the amide I region. The dashed line corresponds to the original spectrum, the blue area indicates the contribution of the inter-molecular β-sheet signal to the total area upon Gaussian deconvolution. (**D**) CR absorbance spectrum in the absence (dashed line) and in the presence (solid line) of TIA1 peptide. (**E**) Fluorescence emission spectrum of Th-T in the absence (dashed line) and in the presence (solid line) of TIA1 peptide. (**F**) TIA1 peptide stained with Th-S and observed at 40X magnification using fluorescence microscopy. (**G**) TIA1 peptide representative transmission electron micrograph. The data in panels C to G were collected upon incubation of TIA1 peptide for 2 days in 5 mM potassium phosphate buffer pH 7.4 at 37 °C.

TIA1 (Fig. 7A) is an RNA-binding protein and a component of stress granules required to regulate alternative splicing and mRNA translation and turnover. The predicted PrLD lies in a Q-rich region. TIA1 has already been suggested to be a functional prion-like protein whose dysregulation is involved in Amyotrophic Lateral Sclerosis (ALS)^33^. However, the presence of an amyloid core inside its PrLD has not been addressed before.

To further confirm the presence of PrLDs in these proteins and to define more precisely their boundaries we used PLAAC, yet another composition-based predictor, in which, in contrast to PAPA, the length of the predicted PrLD also depends on the protein composition. PLAAC detected PrLDs, overlapping with the regions previously identified by PAPA, in all the above described proteins (Figs 1A to 7A).

The predicted soft amyloid cores for these proteins are shown in Table 2. Remarkably, well-validated aggregation predictors like Aggrescan^54^, Tango^55^ and Zyggregator^56^ failed to classify these stretches as aggregation-prone, the exception being the PHC1 core, which was identified by Tango (Table S2). The underlying reason explaining why these amyloid predictors fail to score properly the putative cores is likely their much lower hydrophaticity, when compared with the amyloid stretches present in pathogenic proteins like Aβ42 or α-synuclein (Table S3). Indeed, the predicted cores are enriched in polar residues like Gln, Asn and Ser as well as in Gly (Table 2), all amino acids considered conferring low aggregation propensities to protein sequences.

An analysis of the structural context in which these soft amyloid cores are embedded in their respective sequences using the prediction algorithms FoldIndex^57^, IUPRED^58^, PondR-FIT^59^ and RONN^60^ indicates that they are preferentially located in disordered protein segments (Table S4).

### Predicted human PrLD soft amyloid cores assemble into β-sheet rich structures

We synthetized 21-residues long peptides correspondent to the detected amyloid cores and analyzed their secondary structure content by far-UV circular dichroism (CD) immediately after their dilution at 100 μM in sodium phosphate buffer at pH 7.4 (Figs 1B to 7B). Five out of the seven peptides (DDX5, EYA1, ILF3, MED15 and TIA1) exhibited spectra consistent with a mostly disordered conformation. In ILF3, the high content in Tyr residues (23%) renders a characteristic aromatic signal at 230 nm. The other two peptides (NCOA2 and PHC1) already exhibited a β-sheet CD spectrum immediately after dilution, thus suggesting that they experience a very fast assembly in aqueous buffer.

Next, we incubated the peptides at 100 μM for 2 days at 37 °C and monitored their ability to form macromolecular structures using synchronous light scattering, bis-ANS binding, infrared spectroscopy (ATR-FTIR) and far-UV CD.

The formation of high-order assemblies after incubation was confirmed for all peptides by measuring the light scattering of the correspondent solutions. All of them exhibited significant scattering signal after 2 days (Figure S1). Despite the amyloid cores in this study are less hydrophobic than those of pathogenic amyloids (Table S3), they still exhibit non-polar residues that might contribute to the initial assembly. We explored the presence of exposed hydrophobic clusters in the detected aggregated material by measuring their binding to bis-ANS (Figure S2A), a dye that increases its fluorescence emission upon interaction with these regions^61^. The bis-ANS fluorescence emission maximum increases and blueshifts from 530 nm in the absence of peptides to 510 nm in their presence, indicating the existence of hydrophobic-patches in the surface of all these assemblies. The binding of these assemblies to bis-ANS was, however, much lower than those of the amyloid fibrils formed by the Parkinson’s associated α-synuclein protein at the same concentration (Figure S2B), consistent with the lower hydropathicity of PrLDs amyloid stretches.

Next, we recorded the amide I region of the FTIR spectrum (1700–1600 cm^−1^) for these aggregates (Figs 1C to 7C). This region corresponds to the absorption of the carbonyl peptide bond group of the protein main chain and it is conformation sensitive. Deconvolution of the spectra allowed us to assign the individual secondary structure elements of incubated peptides and their relative contribution to the main absorbance (Table S5). In all the cases we could identify a strong band at 1620–1630 cm^−1^, usually assigned to the presence of inter-molecular β-sheets. This signal is the largest contributor to the absorbance spectrum in all peptides, except for DDX5, where it contributes 37% of the area. Interestingly, no anti-parallel β–sheet band was detected (~1690 cm^−1^) in any of the samples; thus suggesting that the detected β–strands in self-assembled peptides would adopt preferentially a parallel disposition. The other detected structural elements are associated with disordered structure and turns (Table S5). We also monitored the secondary structure of the incubated peptides using far-UV CD (Figure S3). In all cases we could detect a band at 215–220 nm consistent with the population of a β-sheet enriched conformation, despite in some cases the ellipticity was low, indicating that a significant proportion of the peptide was aggregated, therefore out of the solution and not detectable.

Overall, our data are consistent with the spontaneous assembly of the predicted human PrLD amyloid cores into supramolecular β-sheet enriched structures.

### Predicted human PrLD amyloid cores form non-toxic amyloid-like fibrillar structures

We used the amyloid-specific dyes Congo red (CR), Thioflavin T (Th-T) and Thioflavin-S (Th-S) to confirm that the detected β–sheet enriched aggregates were organized into amyloid-like suprastructures.

The absorbance spectra of CR red shifts in the presence of amyloid aggregates^62^. Incubation of CR in the presence of aggregated peptides resulted in a red shift of its spectrum in all the cases (Figs 1D to 7D); despite for DDX5, EYA1 and ILF3 the spectral shift was small. To confirm the ability of these three peptides to bind CR they were incubated under the same conditions at 500 μM final concentration. The peptides in these three solutions promoted a clear shift of the absorption maximum of the die (Figure S4).

Th-T fluorescence emission is enhanced in the presence of amyloid fibrils^63^. All the peptides promoted an increase in the intensity of Th-T fluorescence spectral maximum at 488 nm (Figs 1E to 7E). Furthermore, binding of Th-S to the aggregates could be visualized by fluorescence microscopy for all incubated peptides. Areas rich in fibrous material were stained with Th-S to yield green-yellow fluorescence against a dark background in all cases (Figs 1F to 7F).

The dye binding results indicate that incubated peptide solutions might contain detectable amounts of amyloid-like structures. To confirm this extent, the morphological features of the peptide assemblies in these samples were analyzed using transmission electron microscopy (TEM). Negative staining indicated that all peptides effectively assemble into supramolecular structures (Figs 1G to 7G). The aggregates formed by DDX5, EYA1, MED15, NCOA2, and TIA1 correspond to amyloid-like fibrillar arrangements, without any significant accumulation of amorphous material. The fibrils exhibit a diameter that varies from 5 to 10 nm and a length that ranges from 2 to 10 μm. In the ILF3 sample long amyloid-like fibrils coexist with small aggregates that appear to attach to the fibrils, whereas, for PHC1, despite its amyloid-like tintorial properties, the material appears to be essentially protofibrillar. All the peptides were also able to form macromolecular aggregates when they were incubated at 10 μM (1/10 the concentration used in the previous assays) (Figure S5).

The above data indicate that the predicted amyloid cores exhibit a strong propensity to form amyloid-like assemblies. The amyloids formed by pathogenic protein fragments are usually highly cytotoxic. We tested if the aggregates formed by the PrLD amyloid cores display any toxicity when administered to neuroblastoma SH-SY5Y cells. All the aggregates were essentially innocuous when added to the cell cultures at up to 10 μM (Figures S6).

### Predicted human PrLD amyloid cores form aggregates with self-seeding properties

**Figure 8:**
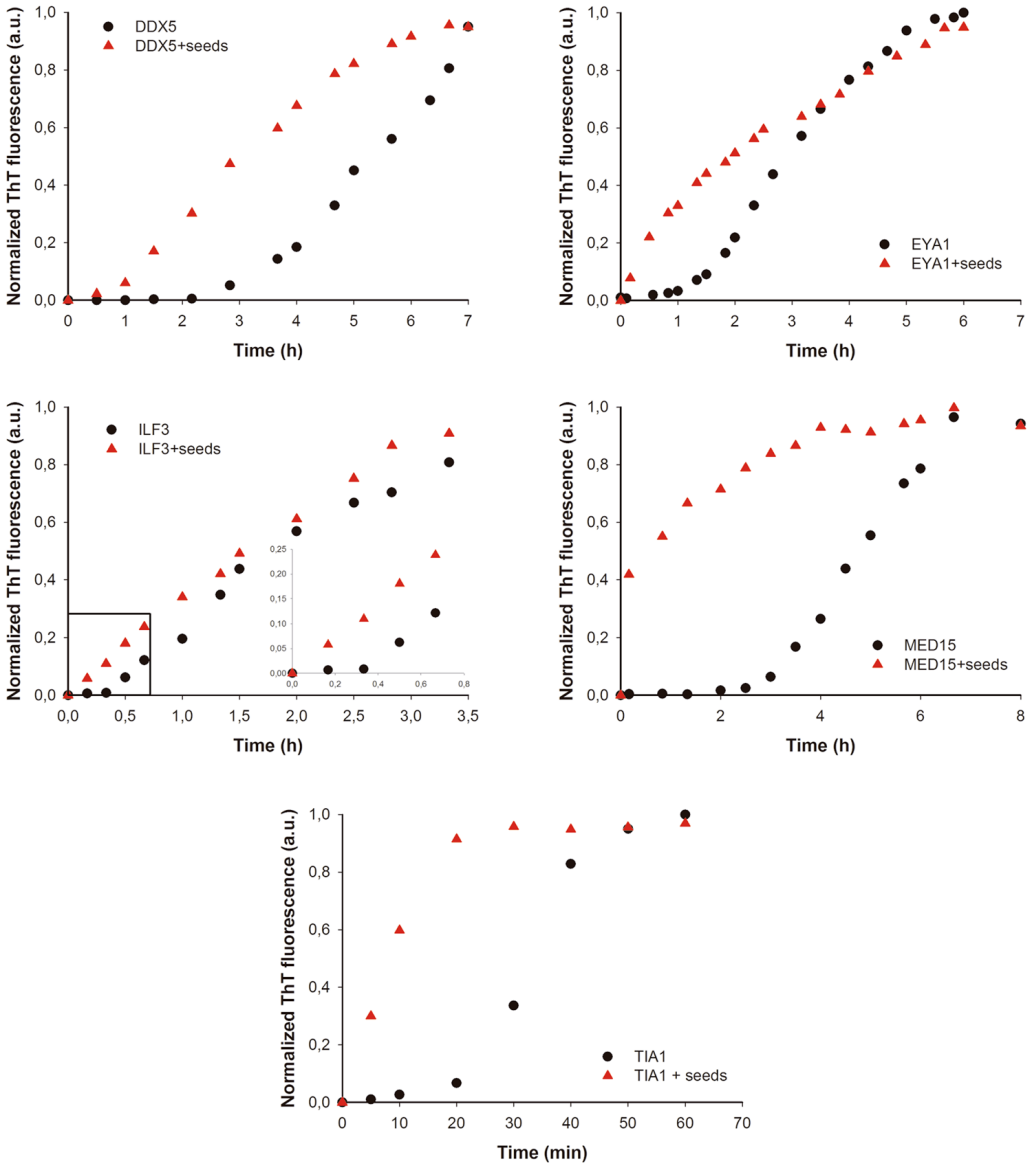
Human PrLD amyloid cores kinetics. Aggregation kinetics of DDX5, EYA1, ILF3, MED15 and TIA1 peptides at 100 µM in the absence (black circles) and presence (red triangles) of 2% (w/w) of preformed aggregates (seeds) were monitored by tracking the changes in Th-T fluorescence emission spectra. All the peptides exhibited accelerated kinetics in the presence of seeds.

Seeded protein aggregation is a well-established mechanism for *in vivo* amyloid fibril formation and underlies prion propagation^64^. The nucleation step of the amyloid assembly is shortened in the presence of preformed amyloid fibrils of the same protein, that can act as nuclei for the subsequent polymerization reaction^65^. Specific and short aggregation-prone regions have been shown to play a crucial role in this process^66,67^. To test whether preformed PrLDs core amyloid-like assemblies can seed the aggregation of the correspondent soluble peptides, we followed the aggregation kinetics of the peptides at 100 µM in the presence and absence of 2% (w/w) of preformed aggregates (Fig. 8). We could not monitor the aggregation kinetics of NCOA2 or PHC1 because they exhibited very high Th-T signal from the very beginning of the reaction, consistent with a very fast assembly into β-sheet structures, as suggested by the far-UV CD spectra they exhibit immediately upon dilution in aqueous solution (Figs 5B and 6B). The rest of peptides exhibited characteristic sigmoidal aggregation kinetics with lag-phases ranging from 20 to 120 min in the absence of seeds. The addition of preformed aggregates strongly accelerated the formation of Th-T positive assemblies in all cases (Fig. 8), supporting a nuclei-dependent aggregation mechanism and raising the possibility that such specific interactions could also occur in the context of the complete proteins in which these short regions are embedded.

## Discussion

The prion phenomenon is best known by its association with spongiform encephalopaties in mammals, but in the last years a growing list of non-pathological prion-like proteins are being discovered. Functional prions were originally identified in yeast^68^. In these prion proteins, specific PFDs encode for their ability to switch between soluble and self-assembled states. Computational analysis are revealing the existence of polypeptides displaying similar domains in previously unexplored proteomes^17^. This suggests that this type of conformational conversion might be an evolutionary conserved mechanism exploited by different organisms, including humans, for beneficial purposes.

A characteristic feature of many pathogenic amyloids is the presence of short hydrophobic sequence stretches able to nucleate the assembly of these proteins into toxic aggregates both *in vitro* and *in vivo* under pathological conditions. These highly amyloidogenic regions seem to be absent in PFDs and PrLDs, likely because their potency will unbalance the equilibrium between the soluble and self-assembled states of the proteins towards an irreversibly aggregated and potentially toxic state, even in the absence of stress. However, we have recently proposed that in addition to a special compositional bias, PFDs and PrLDs contain indeed cryptic soft amyloid cores that can play an important role at the early stages of assembly by restricting inter-molecular interactions to specific regions of these long domains^28^. In these cores, the amyloid nucleating potency would be weaker and less concentrated that in pathogenic amyloids. This property would allow the protein to remain soluble in most physiological conditions, while being responsive to conditions that favor inter-molecular contacts or to the presence of preformed assemblies that target these specific segments. We have provided evidences for the presence of such regions in the PFDs of four of the best characterized yeast prions and for the ability of one of these soft amyloid cores to recruit the assembly of its correspondent full-length PFD *in vitro* and in the cell^30^.

Using an approach analogous to the one described here, we previously screened the *C. Botulinum* proteome for the presence of regions displaying both compositional similitude to *bona fide* prions and containing a soft amyloid core, identifying a first bacterial PrLD in the Rho Terminator factor of this pathogen^31^. Later on, Yuan and Hochschild validated the prion-like nature of this protein, demonstrating that Rho can access alternative protein conformations in prokaryotes, including a self-assembled state with decreased Rho activity that results in genome-wide changes at the transcriptome level^32^. The seven human proteins selected in the present study for experimental characterization, exhibit predicted PrLDs with the same compositional properties than Rho or the yeast prions and are as well regulatory nucleic acid-binding proteins. We show here that they all contain a region able to autonomously self-assemble into amyloid-like structures displaying low cytotoxicity, a property that is likely linked to the weak hydrophobic patches in these assemblies, compared with those present in the fibrils of pathogenic proteins like α-synuclein^1^. In addition, for all the cases we could test, the fibrillar assemblies had the capacity to seed and accelerate the aggregation reaction of their soluble counterparts. Despite our assays are *in vitro* and just with a piece of the protein, the results reveal the self-assembly potential encoded in human prion-like proteins.

Similar to Short Linear Interaction Motifs (SLIMs) in intrinsically disordered proteins, the assembling properties of soft amyloid cores might contribute to mediate PrLDs functional protein-protein interactions (PPI)^28^. Actually, it is known that the interaction between PrLDs of RNA granules proteins like TDP43, TAF15 and FUS is sufficient to induce liquid-liquid phase separation^7^, but also reversible hydrogels^69^, which have a high β-sheet content, but are labile to dilution. Interestingly enough, mutations that severely impede the formation of intermolecular β-sheets also inhibit the ability of PrLDs to activate transcription, indicating that, in this particular context, the formation of soft amyloid-like assemblies plays a functional role^70^. DDX5, ILF3 and TIA1 are also RNA granule proteins^43,71–73^; thus it is tempting to propose that the identified PrLDs and embedded soft amyloid cores might likewise play a role in the formation of functional β-sheet-containing assemblies. The other four proteins in our study are either structural components of macromolecular complexes (MED15 and PHC1) or they are involved in protein-protein interactions (NCOA2 and EYA1). The detected PrLDs and the sticky nature of their cores might facilitate these contacts. It has been suggested that the role of PrLDs would be to promote a primary level of organization through homotypic interactions and that, once oligomerized, these assemblies would facilitate the establishment of novel lateral interactions with other proteins^74^. Indeed, an analysis of the interaction networks of the seven proteins in this study using the STRING PPI network database^75^ indicates that, as a trend, they tend to establish more PPI than the average human proteome (Table S6).

From an evolutionary point of view, the fact that the detected amyloid cores have been not purged out by natural selection support their functional role, since there is a strong selective pressure to reduce the amyloidogenic load of protein sequences, especially when they are located in disordered regions and thus exposed to solvent^76^. The presence of this kind of regions is inherently risky, since mutations that would increase their amyloid potency, making them more similar to classical pathogenic amyloid stretches, can promote irreversible protein aggregation, generating a deleterious phenotype resulting from deregulation of the homo or heterotypic PPIs in which they are involved. This is the case of hnRNPA1 and hnRNPA2, two human prion-like RNA-binding proteins in which point mutations mapping exactly in the pWALTZ predicted soft amyloid core increase the aggregation propensity of their PrLDs, causing the loss of their regulatory activity and leading to the onset of multisystem proteinopathy and ALS^14,27^. It is worth to explore whether the link to disease of the nucleic acid-binding human proteins in our subproteome and specifically of the seven polypeptides we studied here owes to a related mechanism. This would imply that a common process of mutation driven miss-assembly of cryptic amyloid cores in PrLDs might account for very different pathological phenotypes, from cancer to neurodegenerative disorders, depending on the affected protein and the pathways it regulates.

## Methods

### Computational identification of PrLDs in human proteins

The human reference proteome dataset was downloaded from Uniprot^77^ and scanned for PrLDs using PAPA^12^ with the default parameters. From the initial 70940 proteins in the proteome, 663 prion-like candidates were identified. Their putative PrLDs were further evaluated with pWALTZ^14^ with a threshold of 65 in order to identify those domains containing a soft amyloid core, which resulted in 535 final positive predictions. The protein sequences selected for experimental characterization were also analyzed with PLAAC^16^ to define the boundaries of their PrLDs.

### Peptide preparation

We obtained the sequence of the 21 amino acid core region as predicted by pWALTZ^14^ for the seven protein candidates selected for experimental characterization. The correspondent peptides were purchased from CASLO ApS (Scion Denmark Technical University). The lyophilized peptides were solubilized at a final concentration of 5 mM in dimethyl sulfoxide (DMSO) or in hexafluoro-2-propanol for CD analysis, in order to avoid the large increase in voltage caused by residual DMSO. Right before each experiment, the stock solutions were diluted to 100 μM in 5 mM sodium phosphate buffer pH 7,4. For aggregation assays the samples were incubated for 2 days at 37 °C with continuous agitation at 150 rpm in the presence of Teflon beads.

### Synchronous light scattering

Synchronous light scattering was monitored using a JASCO Spectrofluorometer FP-8200. The conditions of the spectra acquisition were: excitation wavelength of 360 nm, emission range from 350 to 370 nm, slit widths of 5 nm, 0.5 nm interval and 1000 nm/min scan rate. The peptides were sonicated for 10 min in an ultrasonic bath (Fisher Scientific FB15052) before measurement. 100 μl of peptide solution was analyzed.

### Bis-ANS (4,4-Dianilino-1,1-binaphthyl-5,5-disulfonate) binding

The fluorescent spectrum of bis-ANS was analyzed using a JASCO Spectrofluorometer FP-8200. The conditions of the spectra acquisition were: excitation wavelength of 365 nm, emission range from 440 to 640 nm, slit widths of 5 nm, 0,5 nm interval and 1000 nm/min scan rate. The peptides were sonicated for 10 min in an ultrasonic bath (Fisher Scientific FB15052) before dye addition. 10 µl of peptide solution was added to 100 µl of 10 µM bis-ANS in H_2_O. A 10 µM bis-ANS solution without peptide was used as a control.

### Circular dichroism (CD) spectroscopy

CD experiments were performed using a JASCO J-715 spectropolarimeter. Measurements of the far-UV CD spectra (260–190 nm) were made by the addition of 200 µl of the sample to a cuvette of 0.1 cm path-length. Spectra were recorded at room temperature, 1 nm band width and 100 nm/min scan rate. The resulting spectrum was the average of 10 scans. The contribution of the buffer was subtracted.

### Fourier transform infrared (FT-IR) spectroscopy

FTIR experiments were performed using a Bruker Tensor 27 FT-IR spectrometer (Bruker Optics Inc) with a Golden Gate MKII ATR accessory. Each spectrum consists of 16 independent scans, measured at a spectral resolution of 4 cm^−1^ within the 1800–1500 cm^−1^ range. All spectral data were acquired and normalized using the OPUS MIR Tensor 27 software. Data was afterwards deconvoluted using the Peak Fit 4.12 program. The buffer without peptide was used as a control and subtracted from the absorbance signal before deconvolution.

### Transmission electron microscopy (TEM)

The morphology of the aggregated peptides was evaluated by negative staining and using a JEOL JEM-1400Plus Transmission Electron Microscope. 5 µl of peptide solution was placed on carbon-coated copper grids and incubated for 5 min. The grids were then washed and stained with 5 µl of 2% w/v uranyl acetate for 5 min. Then, grids were washed again before analysis.

### Congo red (CR) binding

CR binding to aggregated peptides was analyzed using a Specord® 200 Plus spectrophotometer (Analyticjena). The absorbance spectra were recorded from 400 to 650 nm. Spectra were acquired at 50 nm/sec scan rate. Peptides were sonicated for 10 min in an ultrasonic bath (Fisher Scientific FB15052) before dye addition. 10 μl of the sonicated aggregated peptide was added to 100 µl of 5 µM CR in 5 mM sodium phosphate buffer pH 7,4, and was incubated at room temperature for 5 min before the measurement. The same buffer with 5 µM CR and without peptide was employed as a control.

### Thioflavin-T (Th-T) binding

The fluorescence spectra of Th-T were recorded using a JASCO Spectrofluorometer FP-8200. The conditions of the spectra acquisition were: excitation wavelength of 440 nm, emission range from 460 to 600 nm, slit widths of 5 nm, 0,5 nm interval and 1000 nm/min scan rate. Peptides were sonicated for 10 min in an ultrasonic bath (Fisher Scientific FB15052) before dye addition. 5 µl of the sonicated aggregated peptide was added to 100 µl of 25 µM ThT in 5 mM sodium phosphate buffer pH 7,4. The same buffer with 25 µM ThT and without peptide was employed as a control.

### Thioflavin-S (Th-S) staining

First, 150 µl of aggregated peptides were incubated for 1 h in the presence of 125 µM of ThS in 5 mM sodium phosphate buffer pH 7,4. Then, the samples were washed two times with the same buffer. Finally, the precipitated fraction was resuspended in a final volume of 10 µl and placed on a microscope slide and sealed. Images of the peptide aggregates bound to Th-S were obtained at 40-fold magnification in a Leica fluorescence microscope (Leica DMRB).

### Aggregation kinetics and seeding assays

Reactions were carried out at of 100 μM final soluble peptide concentration in a solution containing 25 μM of Th-T at 37 °C in the absence or presence of 2% seeds under quiescent conditions. The aggregation kinetics were followed monitoring the changes in Th-T fluorescence intensity at 488 nm over the time using a JASCO Spectrofluorometer FP-8200. Before each measure, the sample was mixed by pipetting up and down. The conditions of the spectra acquisition were: excitation wavelength of 440 nm, emission range from 460 to 600 nm, slit widths of 5 nm, 0,5 nm interval and 1000 nm/min scan rate. The seeds were prepared by sonicating the preformed aggregates of the corresponding peptide for 10 min in an ultrasonic bath (Fisher Scientific FB15052) before addition.

### Cell viability assay

Human SH-SY5Y cells were seeded into 96-well tissue culture plate with a density of 4,000 cells/well (100 µL/well) in F-12 medium supplemented with 10% FBS, and maintained at 37 °C and 5% CO_2_ atmosphere. Cell cultures were incubated in the presence of different concentrations of peptides for 72 hours. To control cells, the same volume of PBS1x was added. Following incubation, cells were stained by adding 20 µL PrestoBlue® Cell Viability Reagent (Invitrogen) directly to the sample wells. After 30 min of incubation, cell viability was determined by measuring fluorescence exciting at 531 nm and collecting emission at 615 nm in a Victor fluorescent plate reader (Perkin Elmer).

## Electronic supplementary material

Supplementary information
