# Supplementary material for: Characterization of Soft Amyloid Cores in Human Prion-Like Proteins
Source: Sci Rep. 2017 Sep 21;7:12134. doi: 10.1038/s41598-017-09714-z (PMC5608858; doi:10.1038/s41598-017-09714-z)
Supplement: Supplementary file 1 — Supplementary information [file 41598_2017_9714_MOESM1_ESM.pdf]

Supplementary information:

Characterization of Soft Amyloid Cores in Human Prion-Like Proteins

Cristina Batlle[1], Natalia S. de Groot[2,3], Valentin Iglesias[1], Susanna Navarro[1], Salvador Ventura[1]

[1]Institut de Biotecnologia i de Biomedicina and Departament de Bioquímica i Biologia Molecular, Universitat Autónoma de Barcelona, Bellaterra, 08193, Spain
[2]Centre for Genomic Regulation (CRG), The Barcelona Institute for Science and Technology, Dr. Aiguader 88, 08003 Barcelona, Spain.
[3]Universitat Pompeu Fabra (UPF), Barcelona, Spain.

SUPPLEMENTARY FIGURES

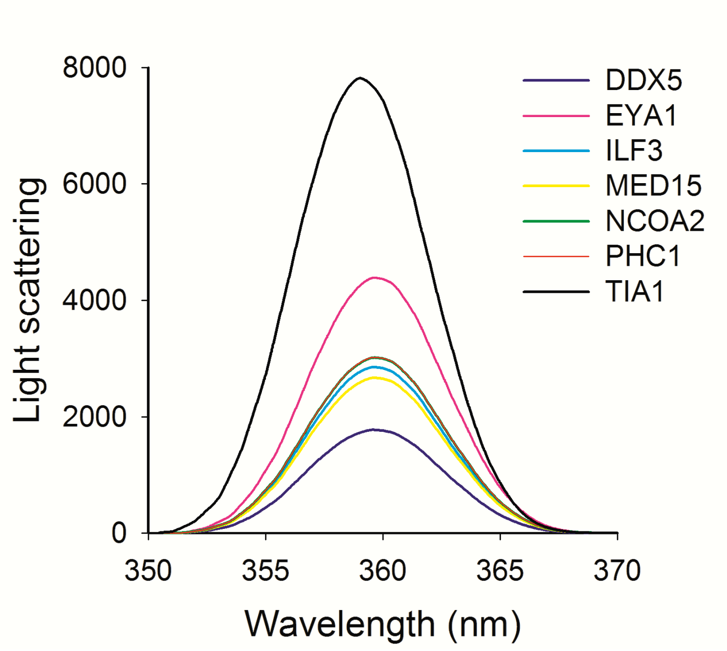

Figure S1. Aggregation of human PrLD amyloid cores. The ability to form macromolecular structures was measured by synchronous light scattering. All selected peptides exhibited a significant scattering signal at 100 µM in 5 mM potassium phosphate buffer at pH 7.4 after incubation at 37 °C for 2 days.

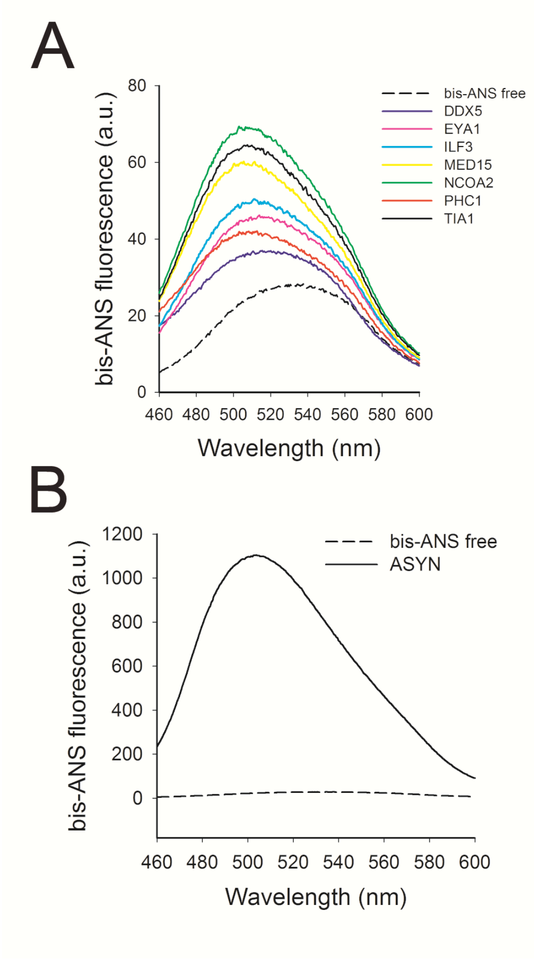

Figure S2. Binding of bis-ANS to aggregated human PrLD amyloid cores. A) Spectra of bis-ANS in the presence of the seven peptides after their incubation at 100 µM in 5 mM potassium phosphate buffer at pH 7.4 and 37 °C for 2 days (color solid lines). The spectrum of free bis-ANS is shown as a dashed line. B) Bis-ANS binding for α-synuclein (ASYN) at 100 µM in 5 mM potassium phosphate buffer at pH 7.4 incubated at 37 °C for 7 days.

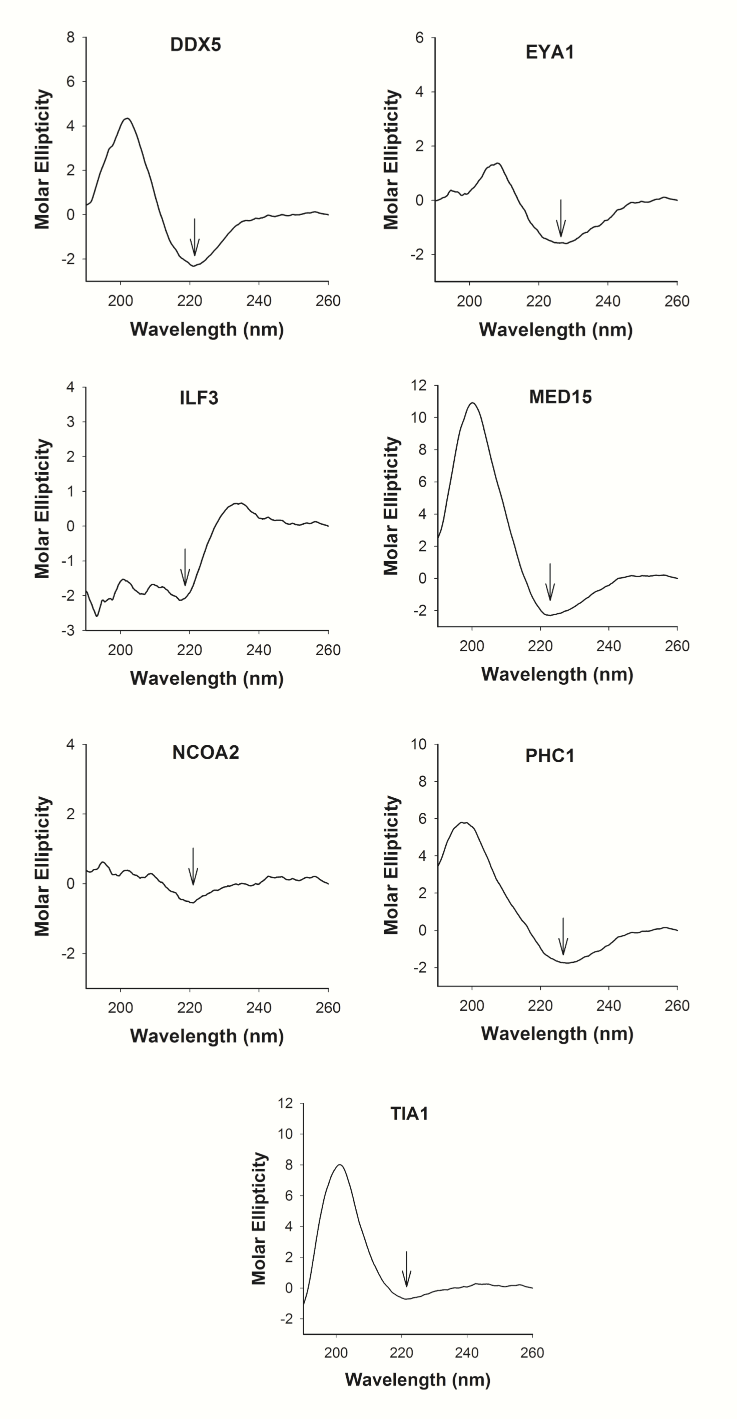

Figure S3. CD spectra of aggregated human PrLD amyloid cores. CD spectra in the far-UV region for peptides incubated at 100 µM in 5 mM potassium phosphate buffer at pH 7.4 and 37 °C for 2 days. The arrow indicates the spectra minima correspondent to β-sheet secondary structure.

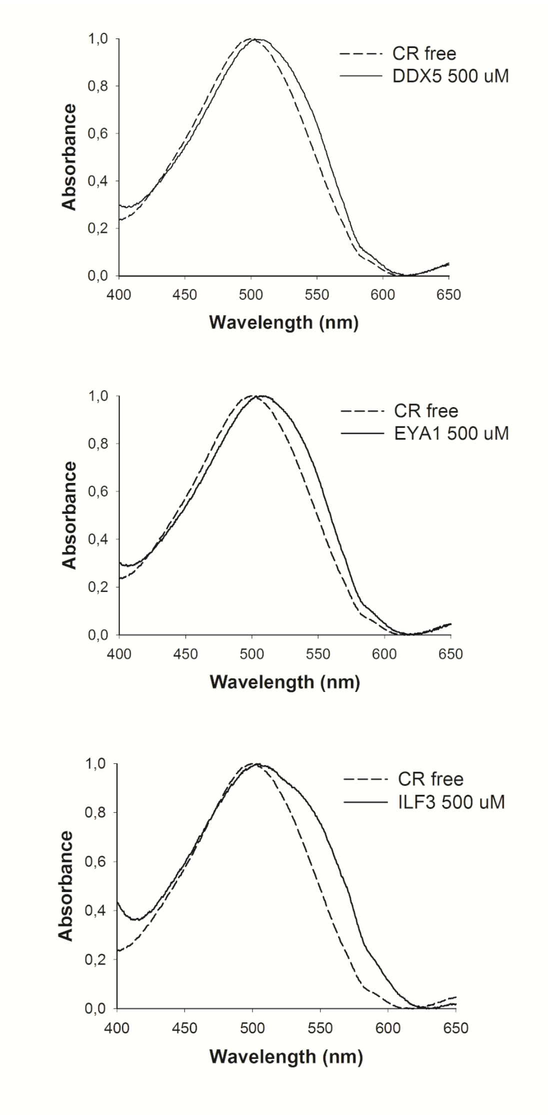

Figure S4. CR binding to DDX5, EYA1 and ILF3 amyloid cores. CR absorbance spectrum in the absence (dashed line) and in the presence (solid line) of DDX5, EYA1 and ILF3 peptides incubated at 500 µM in 5 mM potassium phosphate buffer at pH 7.4 and 37 °C for 2 days.

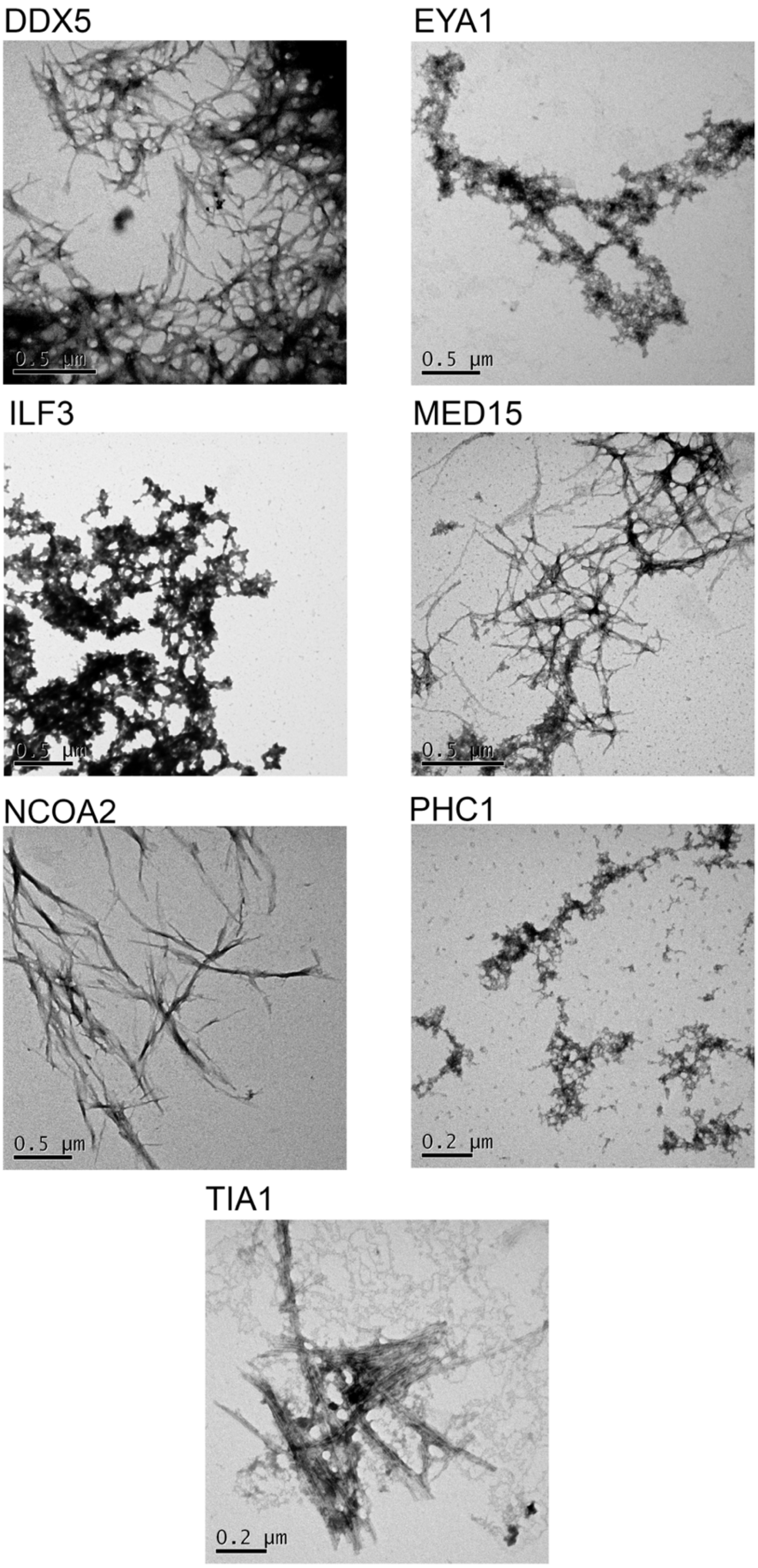

Figure S5. Transmission electron micrographs of peptides incubated at 10 µM. The images were acquired upon incubation of the peptides for 5 days in 5 mM potassium phosphate buffer pH 7.4 at 37 °C.

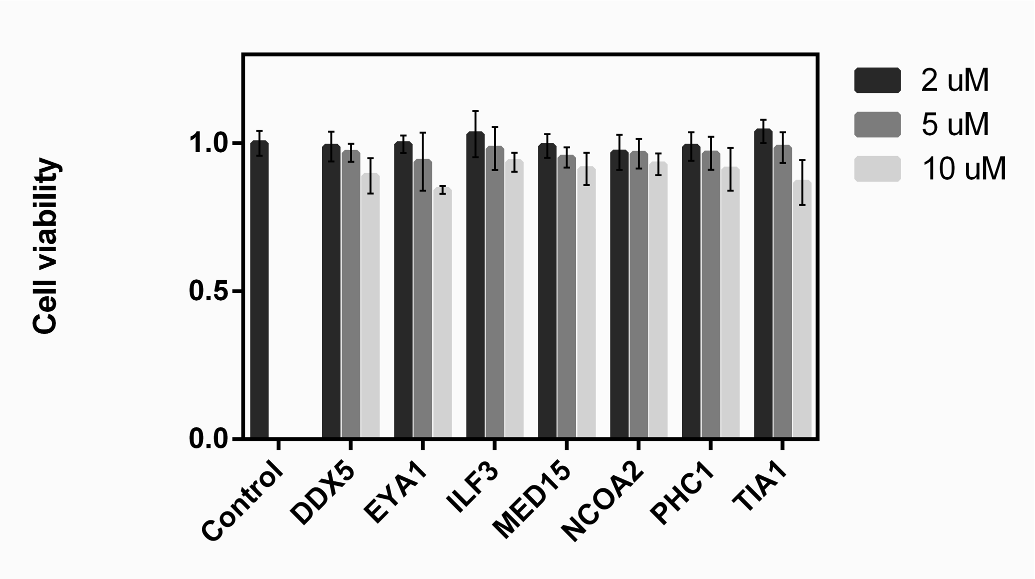

Figure S6. Cytotoxicity of aggregated human PrLD amyloid cores. Cell viability was calculated relative to cells treated with PBS1x buffer (100% viability). The assays were done in five wells for each concentration and the experiment was repeated twice. The data show the averaged values of two independent experiments, and the bars represent the standard error of the mean. Peptides were added at 2, 5 or 10 μM final concentration to SH-SHY cells.

SUPPLEMENTARY TABLES

Table S1. **Human PrLD-containing proteins.** Upon an orthogonal screening of the human proteome with PAPA [1] and pWALTZ [2] we detected 535 human proteins bearing putative PrLDs. For each candidate it is shown its PAPA score, its amyloid core sequence, the pWALTZ score for this protein region and its Ensembl ID according to Uniprot [3].

HUMAN PrLD-CONTAINING PROTEINS				
**Uniprot ID**	**PAPA Score**	**pWALTZ Amyloid Core**	**pWALTZ Score**	**Ensembl ID**
Q9UN88	0,0866361	CLFFVFLSLLEYVYINYLFYS	89,8709	ENSG00000268089
O43300	0,07596259	ITGTMALLFSFFFIIFIVFIS	89,6002	ENSG00000146006
Q9P035	0,06972523	VRFSFFLQIYLIMIFLGLYIN	89,425	ENSG00000074696
H3BRL8	0,06972523	VRFSFFLQIYLIMIFLGLYIN	89,425	ENSG00000074696
H3BPZ1	0,06972523	VRFSFFLQIYLIMIFLGLYIN	89,425	ENSG00000074696
H3BS72	0,06972523	VRFSFFLQIYLIMIFLGLYIN	89,425	ENSG00000074696
Q13936	0,05912466	KSNVFYWLVIFLVFLNTLTIA	88,1191	ENSG00000151067
F5H638	0,05912466	KSNVFYWLVIFLVFLNTLTIA	88,1191	ENSG00000151067
A0A0A0MSA1	0,05912466	KSNVFYWLVIFLVFLNTLTIA	88,1191	ENSG00000151067
A0A0A0MR67	0,05912466	KSNVFYWLVIFLVFLNTLTIA	88,1191	ENSG00000151067
F5H522	0,05912466	KSNVFYWLVIFLVFLNTLTIA	88,1191	ENSG00000151067
F5GY28	0,05912466	KSNVFYWLVIFLVFLNTLTIA	88,1191	ENSG00000151067
E9PDI6	0,05912466	KSNVFYWLVIFLVFLNTLTIA	88,1191	ENSG00000151067
H7BYL8	0,05387899	GYVFFEYQYVDNNIFFEFFIQ	87,4343	ENSG00000164659
A8MWY0	0,05021104	GYVFFEYQYVDNNIFFEFFIQ	87,4343	ENSG00000164659
H7C2N5	0,05021104	GYVFFEYQYVDNNIFFEFFIQ	87,4343	ENSG00000164659
C9JA41	0,05021104	GYVFFEYQYVDNNIFFEFFIQ	87,4343	ENSG00000164659
Q9H7F0	0,05578504	NYENTTVFFISSFQYLIVAIA	86,8609	ENSG00000133657
H7C4S8	0,07745829	KSVTFYWLVIVLVFLNTLTIS	86,4309	ENSG00000157388
Q01668	0,06549277	KSVTFYWLVIVLVFLNTLTIS	86,4309	ENSG00000157388
A0A1B0GUB6	0,06549277	KSVTFYWLVIVLVFLNTLTIS	86,4309	ENSG00000157388
A0A1B0GTN0	0,06549277	KSVTFYWLVIVLVFLNTLTIS	86,4309	ENSG00000157388
A0A1B0GUN6	0,06549277	KSVTFYWLVIVLVFLNTLTIS	86,4309	ENSG00000157388
Q59GD8	0,06429075	KSVTFYWLVIVLVFLNTLTIS	86,4309	ENSG00000157388
A0A1B0GWE1	0,06429075	KSVTFYWLVIVLVFLNTLTIS	86,4309	ENSG00000157388
Q9NX07	0,1563495	YSQMYSYSYNQYYQQYQNYYA	85,2683	ENSG00000180098
Q13324	0,06239437	LSQIMFIYFNSFLQSFQGFFV	84,9976	ENSG00000106113
Q6PJF5	0,06162701	FTYWLTFVHVIITLLVICTYG	84,8224	ENSG00000129667
P34998	0,0566561	FIYFNSFLESFQGFFVSVFYC	84,4879	ENSG00000120088;ENSG00000278232;ENSG00000276191
B3TIK8	0,0566561	FIYFNSFLESFQGFFVSVFYC	84,4879	ENSG00000120088;ENSG00000278232;ENSG00000276191
Q9NR82	0,0714548	RGWAFIYHAFVFLLVFGCLIL	84,0101	ENSG00000185760
Q96G97	0,07405873	ASNFTFLSVIVLFSYMQWVWG	83,7553	ENSG00000168000
J3KQ12	0,05574767	ASNFTFLSVIVLFSYMQWVWG	83,7553	ENSG00000168000
P51861	0,07019652	GYWKTWIFWKTWIFWKTWIFR	83,3572	ENSG00000184258
Q9Y4I1	0,05922493	DIYGFETFEINSFEQFCINYA	83,2138	ENSG00000197535
F8W6H6	0,05922493	DIYGFETFEINSFEQFCINYA	83,2138	ENSG00000197535
G3V394	0,05922493	DIYGFETFEINSFEQFCINYA	83,2138	ENSG00000197535
F8WE88	0,05922493	DIYGFETFEINSFEQFCINYA	83,2138	ENSG00000197535
A0A087WY00	0,05922493	DIYGFETFEINSFEQFCINYA	83,2138	ENSG00000197535
Q9ULS6	0,05845085	LCVFSFSQEIEYWGINEFFID	83,0387	ENSG00000156486
Q6V1P9	0,06386404	LVTFSNIDHDWTRENTYVEYS	82,6405	ENSG00000197410
A0A096LNH0	0,06386404	LVTFSNIDHDWTRENTYVEYS	82,6405	ENSG00000197410
Q96G30	0,05753923	AHKYSIVIGFWVGLAVFVIFM	82,5449	ENSG00000135324
H7BZA3	0,11752418	GQQWIWLQTHYYITYHQWNSK	82,4335	ENSG00000170485
O14979	0,11734311	QGQNWNQGFNNYYDQGYGNYN	82,2742	ENSG00000152795
P35749	0,05757759	DIAGFEIFEVNSFEQLCINYT	82,1149	ENSG00000133392;ENSG00000276480
Q6EIG7	0,05809473	NTEAEQNFIVQQLNESFSYFL	81,892	ENSG00000205846
Q14330	0,11151439	AFTTFLMNLSTCLDVILYYIV	81,876	ENSG00000125245

Q8NEV4	0,07197329	NEQIQYYYNQHVFAWEQNEYL	81,8442	ENSG00000095777
O15516	0,09271811	GQQWIWLQTHYYITYHQWNSR	81,7646	ENSG00000134852
Q9UKN7	0,06258927	FEQLCINYANENLQYLFNKIV	81,7486	ENSG00000091536
A0A087WYA1	0,06258927	FEQLCINYANENLQYLFNKIV	81,7486	ENSG00000091536
Q9UM54	0,08939663	DIAGFEYFEHNSFEQFCINYC	81,7009	ENSG00000196586
E7EW20	0,08939663	DIAGFEYFEHNSFEQFCINYC	81,7009	ENSG00000196586
A0A0A0MRM8	0,08939663	DIAGFEYFEHNSFEQFCINYC	81,7009	ENSG00000196586
A0A0D9SGC1	0,08939663	DIAGFEYFEHNSFEQFCINYC	81,7009	ENSG00000196586
O95178	0,06500774	QVFQSEFFSGLMWFWILWRFW	81,6212	ENSG00000090266
H7C5B8	0,06500774	QVFQSEFFSGLMWFWILWRFW	81,6212	ENSG00000090266
C9IZW8	0,05916781	QVFQSEFFSGLMWFWILWRFW	81,6212	ENSG00000090266
C9JXM4	0,05857762	QVFQSEFFSGLMWFWILWRFW	81,6212	ENSG00000090266
Q13459	0,10386017	RNSFEQFCINYANEQLQYYFN	81,4779	ENSG00000099331
M0R0P8	0,10386017	RNSFEQFCINYANEQLQYYFN	81,4779	ENSG00000099331
M0R300	0,10386017	RNSFEQFCINYANEQLQYYFN	81,4779	ENSG00000099331
Q8N119	0,09105683	GEVMVRFSTYFFRNSWYWLYE	81,4142	ENSG00000154485
Q8NHU2	0,0599964	NEMDIEYIRSHYNIEDFIYFS	81,3983	ENSG00000089101
P42261	0,06674154	AYEIWMCIVFAYIGVSVVLFL	81,1594	ENSG00000155511
P42262	0,06542405	AYEIWMCIVFAYIGVSVVLFL	81,1594	ENSG00000120251
F8W7L6	0,06542405	AYEIWMCIVFAYIGVSVVLFL	81,1594	ENSG00000120251
E7EWC7	0,16652972	AEASALQQQQYYQWYQQYNYA	81,1434	ENSG00000167615
A0A0G2JPW6	0,16652972	AEASALQQQQYYQWYQQYNYA	81,1434	ENSG00000276681
A0A087WUE4	0,16652972	AEASALQQQQYYQWYQQYNYA	81,1434	ENSG00000167615
Q96PV6	0,12227261	AEASALQQQQYYQWYQQYNYA	81,1434	ENSG00000167615;ENSG00000276681;ENSG00000276458;ENSG00000274305
A0A087WTE7	0,12227261	AEASALQQQQYYQWYQQYNYA	81,1434	ENSG00000274305;ENSG00000276681;ENSG00000167615;ENSG00000276458
C9JMY0	0,12227261	AEASALQQQQYYQWYQQYNYA	81,1434	ENSG00000167615
A0A0G2JN70	0,12227261	AEASALQQQQYYQWYQQYNYA	81,1434	ENSG00000276681
Q9UKF5	0,0936033	WWIHFRIVEIVVVIDNYLYIR	81,1116	ENSG00000168594
Q9Y6U3	0,07828965	VDQNSYGEFYGGDCYIILYTY	81,0957	ENSG00000006747
Q9UL59	0,05085828	QEEKFRYLEYENFSYWQGWWN	81,0638	ENSG00000149050
E3W988	0,06579112	YTVHLKQRYFLADNFMIYLYN	80,809	ENSG00000197140;ENSG00000275594
Q8WXR4	0,13605077	RNSFEQLCINIANEQIQYYFN	80,7134	ENSG00000071909
F5H2J1	0,13605077	RNSFEQLCINIANEQIQYYFN	80,7134	ENSG00000071909
A0A1B0GUS7	0,06123439	DGQYIYSLLTDSTGQYAYLFI	80,602	ENSG00000198722
A4D1P6	0,05156147	YDENTVYSIGEDGKFIQWNIH	80,5701	ENSG00000105875
C9J1X0	0,05156147	YDENTVYSIGEDGKFIQWNIH	80,5701	ENSG00000105875
Q6PIF6	0,09351191	DIFGFENFENNSFEQLCINFA	80,5223	ENSG00000169994
Q9BVK6	0,05865928	QRVLWWSILQTLILVAIGVWQ	80,5223	ENSG00000184840
Q9BQ31	0,06148551	LCVFSFCQEIEYWGINELFID	80,4268	ENSG00000170745
Q9H114	0,0754019	STLNFFIQSYNNASNDTYLYR	80,3949	ENSG00000125823
P10643	0,05645944	FQVKINNDFNYEFYNSTWSYV	80,379	ENSG00000112936
Q8WW62	0,0607709	IQSNYNYVNWWSTAQSLVIIL	80,2675	ENSG00000157315
Q16099	0,10851645	ARVLNSNYAFLLESTMNEYYR	80,156	ENSG00000149403
Q8IXK2	0,05518623	GHQVILYLCHGMGQNQFFEYT	80,1242	ENSG00000119514
Q02817	0,06500462	FDGLYYSYQGNCTYVLVEEIS	80,0605	#N/A
A0A0G2JR65	0,06500462	FDGLYYSYQGNCTYVLVEEIS	80,0605	ENSG00000278466
Q86WI1	0,05742728	TNISYTSTFYGFKEEDYVIIS	79,9012	ENSG00000205038
Q8NDX9	0,12844553	QYISYRCQEKRNTYFAEYWYQ	79,8375	ENSG00000240053;ENSG00000239285;ENSG00000241132;ENSG00000240433;ENSG00000241713;ENSG00000244672;ENSG00000239497
H0Y6P8	0,12844553	QYISYRCQEKRNTYFAEYWYQ	79,8375	ENSG00000263020;ENSG00000258589;ENSG00000258543
A0A0G2JM12	0,12844553	QYISYRCQEKRNTYFAEYWYQ	79,8375	ENSG00000224774;ENSG00000206406
N0E472	0,12844553	QYISYRCQEKRNTYFAEYWYQ	79,8375	ENSG00000258589;ENSG00000263020;ENSG00000228875;ENSG00000224398;EN

				SG00000258543
A0A140T9M8	0,12844553	QYISYRCQEKRNTYFAEYWYQ	79,8375	ENSG00000241713
A0A140T9C1	0,12844553	QYISYRCQEKRNTYFAEYWYQ	79,8375	ENSG00000241132
A0A140T8X3	0,12844553	QYISYRCQEKRNTYFAEYWYQ	79,8375	ENSG00000239285
A0A140T990	0,12844553	QYISYRCQEKRNTYFAEYWYQ	79,8375	ENSG00000239497
P35499	0,05836608	VMILTVFCLSVFALVGLQLFM	79,7738	ENSG00000007314
J3QQZ1	0,05836608	VMILTVFCLSVFALVGLQLFM	79,7738	ENSG00000007314
H0YBY8	0,05675816	QNSEFYGFSEFYYCTEDVLRM	79,6305	ENSG00000197217
Q8N660	0,05879675	QHYRSVFYSFEEEHISFALYV	79,5668	ENSG00000266338
Q9UHI6	0,08907492	TYQDYEEYWRAYYRAWQEYYA	79,5349	ENSG00000064703
E9PJ60	0,08907492	TYQDYEEYWRAYYRAWQEYYA	79,5349	ENSG00000064703
Q8N699	0,07278373	FTVSMAIGLVLGGFIWAVFIC	79,4871	ENSG00000120279
H0YDV5	0,07278373	FTVSMAIGLVLGGFIWAVFIC	79,4871	ENSG00000120279
Q8N9R8	0,05212206	YHYYLRTSETSYLNEAFSFYS	79,4553	ENSG00000173611
Q3SXZ0	0,05212206	YHYYLRTSETSYLNEAFSFYS	79,4553	ENSG00000173611
H3BTA9	0,06513198	MLFQLMVEHDHETFWLFQFFL	79,4075	ENSG00000167139
Q8N7C4	0,11650313	RTVMHCFWMFFVINYAHITYK	79,3916	ENSG00000172738
P27487	0,0553414	QENNILVFNAEYGNSSVFLEN	79,3916	ENSG00000197635
F8WE17	0,0553414	QENNILVFNAEYGNSSVFLEN	79,3916	ENSG00000197635
B2RTY4	0,05122702	DIFGFEDYENNSFEQFCINFA	79,3119	ENSG00000066933
H3BMM1	0,05122702	DIFGFEDYENNSFEQFCINFA	79,3119	ENSG00000066933
H3BRD5	0,05122702	DIFGFEDYENNSFEQFCINFA	79,3119	ENSG00000066933
Q5QGZ9	0,06335282	YCGYINRLYVQYYHCTYKKRM	79,0253	ENSG00000172322
Q96MH7	0,05231866	GSVFKSEGAYFGNYFTYYSIQ	78,9297	ENSG00000172244
Q6ZMG9	0,07885875	CESMWRFSFYLYVFTYGVRFL	78,8819	ENSG00000172292
P12259	0,08243021	CYTTEFYVAYSSNQINWQIFK	78,8342	ENSG00000198734
A0A0A0MRJ7	0,08243021	CYTTEFYVAYSSNQINWQIFK	78,8342	ENSG00000198734
Q9BXY4	0,08910904	MHLRLISWLFIILNFMEYIGS	78,7227	ENSG00000146374
O43823	0,06285794	YENYNYYGAQNTSVTTGATYS	78,6908	ENSG00000105127
O00219	0,05941801	YFREWLYNSLWFHKHHLWMTY	78,3086	ENSG00000103044
Q8J025	0,08605179	ITRSYRFYHNNTFKAYQFYYG	78,2927	ENSG00000154856
J3KTQ6	0,08605179	ITRSYRFYHNNTFKAYQFYYG	78,2927	ENSG00000154856
O75907	0,10169507	LIWLIFFYWLFHSCLNAVAEL	78,0856	ENSG00000185000
Q16832	0,05704869	HCNNMFAKGVKIFKEVQCYFR	78,0538	ENSG00000162733
Q9Y2K6	0,11023015	CQNVINGQWYEFDDQYVTEVH	78,0219	ENSG00000136878
Q9NPL8	0,07422086	RTAVFVTIFNTVNTSLNVYRN	77,9901	ENSG00000113845
G3XA94	0,07422086	RTAVFVTIFNTVNTSLNVYRN	77,9901	ENSG00000113845
P30988	0,07031394	YVMHSLIHFQGFFVATIYCFC	77,9741	ENSG00000004948
A0A0A0MSQ7	0,07031394	YVMHSLIHFQGFFVATIYCFC	77,9741	ENSG00000004948
A0A0A0MRG0	0,07031394	YVMHSLIHFQGFFVATIYCFC	77,9741	ENSG00000004948
A0A0C4DG16	0,07031394	YVMHSLIHFQGFFVATIYCFC	77,9741	ENSG00000004948
Q9UH17	0,05251703	ARVTIMDYEEFAYCWENFVYN	77,9741	ENSG00000179750
B0QYD3	0,05251703	ARVTIMDYEEFAYCWENFVYN	77,9741	ENSG00000179750
Q9NP73	0,09341816	VLQYYFNLGLQCYYHSYWHSM	77,8308	ENSG00000101901
Q9NZP6	0,05469753	TERKFYTSSTHYYGQETYVRR	77,783	ENSG00000185823
Q6ZS30	0,05927247	SSFFEDFQEYCNSNEWQVYIE	77,7034	ENSG00000144426
Q68BL8	0,08969852	VVYNGAFYYNRAFTRNIIKYD	77,5123	ENSG00000162745
F2Z3N3	0,08969852	VVYNGAFYYNRAFTRNIIKYD	77,5123	ENSG00000162745
Q5JY77	0,06392352	EEVNQEAEEETIFGSWFWVID	77,5123	ENSG00000198932
Q5VYJ5	0,0822926	CTFRFYYHMFGKRIYRLAIYQ	77,4964	ENSG00000204740
U5GXS0	0,0822926	CTFRFYYHMFGKRIYRLAIYQ	77,4964	ENSG00000204740
H0Y8G5	0,17667036	QNWNQGYSNYWNQGYGNYGYN	77,4327	ENSG00000138668
Q14103	0,16396614	QNWNQGYSNYWNQGYGNYGYN	77,4327	ENSG00000138668
Q2NKX8	0,06241224	RNGVIITTYQMLINNWQQLSS	77,4167	ENSG00000186871
B5MDQ0	0,06241224	RNGVIITTYQMLINNWQQLSS	77,4167	ENSG00000186871
Q5TCS8	0,10725238	QHQNWYVIDGFHSKWWVWNEV	77,4008	ENSG00000155085
H7C517	0,10725238	QHQNWYVIDGFHSKWWVWNEV	77,4008	ENSG00000155085
Q8N3X1	0,06385026	MGDWQEVWDENTGCYYYWNTQ	77,3371	ENSG00000109920
Q9Y6F1	0,05656975	TLNQTNIENNNNKFYIIQLLQ	77,3371	ENSG00000041880
C9J9C7	0,05475247	TLNQTNIENNNNKFYIIQLLQ	77,3371	ENSG00000041880
A0A024R2X5	0,05475247	TLNQTNIENNNNKFYIIQLLQ	77,3371	ENSG00000041880
Q9UBI9	0,05394886	MHLQCFYEWESSILVQFNCIG	77,2415	ENSG00000112406
Q8N987	0,08028421	MIYEFWENSSVWNSHLQTNYS	77,1301	ENSG00000123119
Q12809	0,06760619	GSLMYASIFGNVSAIIQRLYS	77,0823	ENSG00000055118
Q86U57	0,06760619	GSLMYASIFGNVSAIIQRLYS	77,0823	ENSG00000055118

Q9UKU9	0,07026607	NGVWYRGGHYRSRYQDGVYWA	77,0345	ENSG00000136859
Q99784	0,0560049	GQVVYNGSIYFNKFQSHIIIR	76,8593	ENSG00000130558
O60469	0,05253284	GYQIGYREYSTGGNFQFNIIS	76,7319	ENSG00000171587
Q8WY19	0,05253284	GYQIGYREYSTGGNFQFNIIS	76,7319	ENSG00000171587
A0A087WUI7	0,05253284	GYQIGYREYSTGGNFQFNIIS	76,7319	ENSG00000171587
Q9BXT5	0,08585683	SAWCVYQYSNSNGNAITQTYQ	76,6364	ENSG00000133863
Q68BL7	0,09373137	YVTNYYYGNSLVEFRNLENFK	76,6204	ENSG00000185585
Q9C0C4	0,05184922	FFGVFQAQWGDMYLSAICEYQ	76,6045	ENSG00000168758
O95677	0,08962113	TAFGQNQYAQYYSASTYGAYM	76,3656	ENSG00000112319
F2Z2Y1	0,08962113	TAFGQNQYAQYYSASTYGAYM	76,3656	ENSG00000112319
E7ESD5	0,08962113	TAFGQNQYAQYYSASTYGAYM	76,3656	ENSG00000112319
E9PLN6	0,08962113	TAFGQNQYAQYYSASTYGAYM	76,3656	ENSG00000112319
Q6ZMW3	0,05828773	CRNNLYYTAGKEVVYFVAGVG	76,2382	ENSG00000214595
K7END1	0,06084251	NGINLALAWSGQEAIWQRVYL	76,2223	ENSG00000108784
O15372	0,05733634	ALLDSQFSYQHAIEESVVLIY	76,1426	ENSG00000147677
B3KS98	0,05733634	ALLDSQFSYQHAIEESVVLIY	76,1426	ENSG00000147677
A0A087WZK9	0,05733634	ALLDSQFSYQHAIEESVVLIY	76,1426	ENSG00000147677
E5RGU4	0,05733634	ALLDSQFSYQHAIEESVVLIY	76,1426	ENSG00000147677
Q6IE37	0,06210967	IAQVQTNLDIFTLLCSSFLTV	76,0471	#N/A
P14735	0,09942826	GWFVYQQRNEVHNNCGIEIYY	76,0152	ENSG00000119912
D6RBZ0	0,12263152	GQSQSWNQGYGNYWNQGYGYQ	75,9993	ENSG00000197451
Q99729	0,11929034	GQSQSWNQGYGNYWNQGYGYQ	75,9993	ENSG00000197451
E9PS35	0,05230367	AIQQYGSETEGVFVITFKNYL	75,9197	ENSG00000166938
Q99590	0,08203334	GESSFTYRAYCTEFIEASEIS	75,8719	ENSG00000139218
A0A0A0MTP7	0,08203334	GESSFTYRAYCTEFIEASEIS	75,8719	ENSG00000139218
P27540	0,05041252	KGQVLSVMFRFRSKNQEWLWM	75,856	ENSG00000143437
B8ZZ71	0,10982166	CWFNGIVEENDSNIWKFWYTN	75,8401	ENSG00000170417
C9IYX5	0,10982166	CWFNGIVEENDSNIWKFWYTN	75,8401	ENSG00000170417
Q969J5	0,07793298	NSSVYFVQYKIMFSCSMKSSH	75,8401	ENSG00000164485
Q6ZP80	0,07502435	CWFNGIVEENDSNIWKFWYTN	75,8401	ENSG00000170417
Q12768	0,08698796	FHRSFEYIQDYVNIYGLKIWQ	75,8082	ENSG00000164961
E7EQI7	0,08698796	FHRSFEYIQDYVNIYGLKIWQ	75,8082	ENSG00000164961
Q6R2W3	0,05627706	IFSWMQTNNSSHWTEFLWFIQ	75,8082	ENSG00000232040;ENSG00000248496
A0A140T9Y6	0,05627706	IFSWMQTNNSSHWTEFLWFIQ	75,8082	ENSG00000248496
Q6ZW05	0,06020776	HHFIQHFLREHYNEWITNIYV	75,7604	ENSG00000244694
Q5GH73	0,14903539	SVWIWQSVIHLLQMGQVWRYI	75,6967	ENSG00000171044
B1AJW0	0,05669377	QTKLYLAMNSEGYLYTSELFT	75,6489	ENSG00000129682
Q92913	0,055885	QTKLYLAMNSEGYLYTSELFT	75,6489	ENSG00000129682
H0YCE8	0,05602707	VQEDYDQAFQYYYQATQFASS	75,633	ENSG00000198730
Q6PD62	0,05176503	VQEDYDQAFQYYYQATQFASS	75,633	ENSG00000198730
Q86UK0	0,06020608	IYNLTGQRVENYLISTANEFV	75,3463	ENSG00000144452
Q9BZC7	0,06005375	GRFYFLYGFVWIQDMMERAII	75,3304	ENSG00000107331
E9PGB2	0,06005375	GRFYFLYGFVWIQDMMERAII	75,3304	ENSG00000107331
J3QSS3	0,06005375	GRFYFLYGFVWIQDMMERAII	75,3304	ENSG00000107331
A0A087WXK5	0,06005375	GRFYFLYGFVWIQDMMERAII	75,3304	ENSG00000107331
H0YD08	0,05357163	SSGHYIAYCRNNLNNLWYEFD	75,3145	ENSG00000077254
A0A0A6YYK7	0,05363945	TSSFNFTITASQVVDSAVYFC	75,2986	ENSG00000211799
P04436	0,05240038	TSSFNFTITASQVVDSAVYFC	75,2986	#N/A
E5RJT0	0,06936255	QSTYYGSFVTRALLDSQFSYQ	75,2508	ENSG00000147677
Q6UXN8	0,08785334	WIQNRESCYYVSEIWSIWHTS	75,2189	ENSG00000197992
Q96L03	0,17039236	RKQYQLTVQVAYYTMMMNLYN	75,1552	ENSG00000162814
Q86VZ5	0,08494212	NQQVLKEASQMNLLARVWWYR	75,1552	#N/A
D3DWC4	0,08494212	NQQVLKEASQMNLLARVWWYR	75,1552	ENSG00000198964
Q92802	0,09794422	SNIFQAQDDSQIQNGYYVNNC	75,0915	ENSG00000244754
D6R968	0,09794422	SNIFQAQDDSQIQNGYYVNNC	75,0915	ENSG00000244754
A0A0B4J276	0,05748387	GEDFTTYCNSSTTLSNIQWYK	75,0756	ENSG00000211806
Q9H159	0,06574854	ISAWYNLSITATEKYNIEQIS	74,996	ENSG00000071991
J3KTP3	0,06574854	ISAWYNLSITATEKYNIEQIS	74,996	ENSG00000071991
Q8IZT6	0,07521763	IRMIIAVTSYKRYLWATVTIQ	74,9163	ENSG00000066279
P0C881	0,06174665	SIYYNQEGTCWYEGDWVQNIK	74,9004	ENSG00000155026
B2RC85	0,06174665	SIYYNQEGTCWYEGDWVQNIK	74,9004	ENSG00000169402
Q05BV3	0,06132981	LFYTQIGEIVYHVAAVGVIYN	74,8367	ENSG00000165521
Q99502	0,10337948	MQGSSFTTSSGIYTGNNSLTN	74,6934	ENSG00000104313
A6NCB9	0,10337948	MQGSSFTTSSGIYTGNNSLTN	74,6934	ENSG00000104313

E7EQM5	0,10337948	MQGSSFTTSSGIYTGNNSLTN	74,6934	ENSG00000104313
F8WB53	0,10337948	MQGSSFTTSSGIYTGNNSLTN	74,6934	ENSG00000104313
P11230	0,0818329	GHQEIHIHEGTFIENGQWEII	74,6297	ENSG00000170175
I3L1T7	0,0818329	GHQEIHIHEGTFIENGQWEII	74,6297	ENSG00000170175
Q9UKJ0	0,05310199	YVNRLFLNWTEGQESGFLRIS	74,5978	ENSG00000121716
C9J8P3	0,05310199	YVNRLFLNWTEGQESGFLRIS	74,5978	ENSG00000121716
V9GYC2	0,05310199	YVNRLFLNWTEGQESGFLRIS	74,5978	ENSG00000121716
C9JNA4	0,05310199	YVNRLFLNWTEGQESGFLRIS	74,5978	ENSG00000121716
P78364	0,07126115	QQQQIHLQQKQVVIQQQIAIH	74,5819	ENSG00000111752
J3KQH6	0,07126115	QQQQIHLQQKQVVIQQQIAIH	74,5819	ENSG00000111752
Q9H2Y7	0,05412621	NHSNSGGGWLSNSGAVDWNHN	74,4863	ENSG00000103994
H3BSS6	0,05412621	NHSNSGGGWLSNSGAVDWNHN	74,4863	ENSG00000103994
Q8N7L0	0,06546473	LLKALNQGQQRYFYSIMRIYN	74,4226	ENSG00000179813
Q8TDW7	0,05700029	ASIVTVIQLVNNVVDTIENEV	74,3908	ENSG00000165323;ENSG00000282908
E9PQ73	0,05700029	ASIVTVIQLVNNVVDTIENEV	74,3908	ENSG00000165323;ENSG00000282908
E9PJL8	0,06251799	RTFAVYLNNTGYRTAFFGKYL	74,3749	ENSG00000137573
Q8IWU6	0,05373492	RTFAVYLNNTGYRTAFFGKYL	74,3749	ENSG00000137573
Q9NRD1	0,07067485	VRYILFQHGGRDTQYWAGWYG	74,3271	ENSG00000116663
J3KQ72	0,07067485	VRYILFQHGGRDTQYWAGWYG	74,3271	ENSG00000116663
H0YNJ6	0,0883015	GMSSEMAMKKYAGGVAEYRYV	74,2634	ENSG00000100938
E5RI03	0,05168671	MRLRFCISSQEYNINNAESFS	74,1997	ENSG00000169398
P04745	0,08106374	VAFGRGNRGFIVFNNDDWTFS	74,1519	ENSG00000174876;ENSG00000187733;ENSG00000237763
P19961	0,08106374	VAFGRGNRGFIVFNNDDWTFS	74,1519	ENSG00000240038
Q9H9S0	0,13721043	QTWNNSTWSNQTQNIQSWSNH	74,0882	ENSG00000111704
Q6NSW7	0,13638297	QTWNNSTWSNQTQNIQSWSNH	74,0882	#N/A
J7H3Z5	0,13638297	QTWNNSTWSNQTQNIQSWSNH	74,0882	ENSG00000255192
Q8N7R0	0,13590225	QTWNNSTWSNQTQNIQSWSNH	74,0882	#N/A
A0A0D9SG05	0,13486792	QTWNNSTWSNQTQNIQSWSNH	74,0882	ENSG00000255192
Q16478	0,0780959	IARVLNSRYAFLLESTMNEYH	74,0882	ENSG00000105737
Q15436	0,05028237	IRVTTIARNWADAQTQIQNIA	74,0086	ENSG00000100934
F5H365	0,05028237	IRVTTIARNWADAQTQIQNIA	74,0086	ENSG00000100934
Q96DX8	0,11143341	AQVQILCHTYWEHWTSQGQVR	73,9448	ENSG00000136514
Q96NE9	0,05192421	RAARYYYYWHLRKQVLHSQCV	73,9448	ENSG00000139926
G3V3V8	0,05192421	RAARYYYYWHLRKQVLHSQCV	73,9448	ENSG00000139926
Q96Q05	0,07622392	SEENFFRIYKRICSVSQISVR	73,8811	ENSG00000167632
P31321	0,05640552	GEWVTNISEGGSFGELALIYG	73,8493	ENSG00000188191
C9JSK5	0,05640552	GEWVTNISEGGSFGELALIYG	73,8493	ENSG00000188191
C9JR00	0,05640552	GEWVTNISEGGSFGELALIYG	73,8493	ENSG00000188191
P13727	0,15379692	LVSIHNFNINYRIQCSVSALN	73,7537	ENSG00000186652
Q9H9V9	0,06168903	TISINHNWVNGFNLANMWRFL	73,7378	ENSG00000081692
A0A087WT84	0,06168903	TISINHNWVNGFNLANMWRFL	73,7378	ENSG00000081692
F8VU51	0,10657491	AAAHWQQHQQHRVGFQYQGIM	73,5785	ENSG00000119596
O94929	0,06945916	SSNVIQCYRCGDTCKGEVVRV	73,2441	ENSG00000173210
D6RHE7	0,06945916	SSNVIQCYRCGDTCKGEVVRV	73,2441	ENSG00000173210
A0A0C4DGA7	0,06945916	SSNVIQCYRCGDTCKGEVVRV	73,2441	ENSG00000173210
Q13117	0,07849245	RRNLWTEAYKWWYLVCLIQRR	73,1804	ENSG00000205944
Q86SG3	0,07849245	RRNLWTEAYKWWYLVCLIQRR	73,1804	ENSG00000205916
Q9NQZ3	0,07849245	RRNLWTEAYKWWYLVCLIQRR	73,1804	ENSG00000188120
E7EU39	0,07849245	RRNLWTEAYKWWYLVCLIQRR	73,1804	ENSG00000205944;ENSG00000205916
E9PBY2	0,07849245	RRNLWTEAYKWWYLVCLIQRR	73,1804	ENSG00000205944
E7EU38	0,07849245	RRNLWTEAYKWWYLVCLIQRR	73,1804	ENSG00000205916
E7ENA5	0,07849245	RRNLWTEAYKWWYLVCLIQRR	73,1804	ENSG00000205916
A0A0A0MSR9	0,07849245	RRNLWTEAYKWWYLVCLIQRR	73,1804	ENSG00000188120
A0A0A0MSS9	0,07849245	RRNLWTEAYKWWYLVCLIQRR	73,1804	ENSG00000205916
Q9NR90	0,07847705	RRNLWTEAYKWWYLVCLIQRR	73,1804	ENSG00000187191
E7ERQ6	0,07847705	RRNLWTEAYKWWYLVCLIQRR	73,1804	ENSG00000187191
E7ETR3	0,07847705	RRNLWTEAYKWWYLVCLIQRR	73,1804	ENSG00000187191
I3L0B6	0,07847705	RRNLWTEAYKWWYLVCLIQRR	73,1804	ENSG00000205944
E7ENA6	0,07847705	RRNLWTEAYKWWYLVCLIQRR	73,1804	ENSG00000205944
A0A140T8Y1	0,07847705	RRNLWTEAYKWWYLVCLIQRR	73,1804	ENSG00000205916

Q92839	0,07410492	QQTRWSKSYFREWLYNALWWH	73,1645	ENSG00000105509
G3V1S7	0,07410492	QQTRWSKSYFREWLYNALWWH	73,1645	ENSG00000105509
M0R2V0	0,07410492	QQTRWSKSYFREWLYNALWWH	73,1645	ENSG00000105509
Q9H3P7	0,08617874	QKQQIMAALNSQTAVQFQQYA	73,0689	ENSG00000182827
P28827	0,06937638	YNVTRCHSYNLTVHYCYQVGG	73,0689	ENSG00000173482
E7EPS8	0,06937638	YNVTRCHSYNLTVHYCYQVGG	73,0689	ENSG00000173482
P38435	0,06425701	HFLTQGYNNWTNGLYGYSWDM	73,053	ENSG00000115486
P31943	0,07854857	SMSGYDQVLQENSSDFQSNIA	72,9574	ENSG00000169045
H0YBK1	0,07854857	SMSGYDQVLQENSSDFQSNIA	72,9574	ENSG00000169045
E9PCY7	0,07292214	SMSGYDQVLQENSSDFQSNIA	72,9574	ENSG00000169045
H0YBG7	0,07292214	SMSGYDQVLQENSSDFQSNIA	72,9574	ENSG00000169045
P07510	0,0519755	FDWQNCSLIFQSQTYSTNEID	72,9415	ENSG00000196811
C9JGE3	0,07807744	YGQQIYFVGSGRTKNGFETRA	72,8778	ENSG00000182944
Q86Y38	0,05531318	RQLQRMFKAIYHKDHFYYIHV	72,7822	ENSG00000103489
Q9NZM6	0,05693646	DLSNFGLQINTEWRYSTSNTN	72,7504	ENSG00000078795
D6RF71	0,05693646	DLSNFGLQINTEWRYSTSNTN	72,7504	ENSG00000078795
Q86X55	0,09376256	VQYFQFYGYLSQQQNMMQDYV	72,7185	ENSG00000142453
K7EQA8	0,09376256	VQYFQFYGYLSQQQNMMQDYV	72,7185	ENSG00000142453
Q12805	0,05409457	ECDASNQCAQQCYNILGSFIC	72,7026	ENSG00000115380
J3QR85	0,07518691	RNGQYVACGCYFSDLQSYYRN	72,5752	ENSG00000011260
Q8N831	0,06318525	IVKVYEVRSFGQVVSFSTLIM	72,5115	ENSG00000178021
P23471	0,06519049	GYVMLMDYLQNNFREQQYKFS	72,4478	ENSG00000106278
Q8NB12	0,05293006	FSMQYISHIFGVINCNGFTLS	72,4478	ENSG00000115593
E9PHG3	0,05293006	FSMQYISHIFGVINCNGFTLS	72,4478	ENSG00000115593
Q9H0B3	0,05081533	SMQAAEEIRILAVITIQAGVR	72,4	ENSG00000130518
A0A087WXN0	0,05081533	SMQAAEEIRILAVITIQAGVR	72,4	ENSG00000130518
Q9UK61	0,10196795	EGENSNSTEQDSYSNFQVYHS	72,2089	ENSG00000163946
A0A087X0F1	0,10196795	EGENSNSTEQDSYSNFQVYHS	72,2089	ENSG00000163946
Q96E52	0,08559848	NHVFFRFNSLSNWRKCNTLAS	72,1611	ENSG00000162600
S4R3A3	0,08559848	NHVFFRFNSLSNWRKCNTLAS	72,1611	ENSG00000162600
X6RDQ1	0,08559848	NHVFFRFNSLSNWRKCNTLAS	72,1611	ENSG00000162600
X6RIG5	0,08559848	NHVFFRFNSLSNWRKCNTLAS	72,1611	ENSG00000162600
X6RL62	0,08559848	NHVFFRFNSLSNWRKCNTLAS	72,1611	ENSG00000162600
X6RD49	0,08559848	NHVFFRFNSLSNWRKCNTLAS	72,1611	ENSG00000162600
Q9Y6X6	0,05021586	QLCVNMTNEKMHHYINEVLFL	72,0496	ENSG00000041515;ENSG00000282848
F8W883	0,05021586	QLCVNMTNEKMHHYINEVLFL	72,0496	ENSG00000041515;ENSG00000282848
Q9NZR2	0,07833707	HQQISHIEHNSRITGMDVYYQ	72,0337	ENSG00000168702
H0Y7T7	0,07833707	HQQISHIEHNSRITGMDVYYQ	72,0337	ENSG00000168702
P35555	0,05446294	GYLQHYQWNQCVDENECLSAH	72,0178	ENSG00000166147
H0YEX9	0,06493815	VERRWMTNYLRLWQLGVEKIY	71,9541	ENSG00000154721
P37088	0,05638715	VNNKRNGVAKVNIFFKELNYK	71,9382	ENSG00000111319
F5GXE6	0,05638715	VNNKRNGVAKVNIFFKELNYK	71,9382	ENSG00000111319
J3QQR9	0,05529633	KILTRNQIETVLSTRIQVMIS	71,8267	ENSG00000213424
M0R010	0,06052653	AGYEGYGYGYGYGQDNTTNYG	71,6356	ENSG00000011243
P17844	0,07038455	TQNGVYSAANYTNGSFGSNFV	71,6196	ENSG00000108654
J3KTA4	0,07038455	TQNGVYSAANYTNGSFGSNFV	71,6196	ENSG00000108654
A0A075B7F2	0,1271674	TDSSYGGQNYSGYSSYGQSYSQ	71,5878	ENSG00000270647;ENSG00000276833
P35557	0,0747984	AMVNDTVATMISCYYEDHQCE	71,5878	ENSG00000106633
C9JQD1	0,0747984	AMVNDTVATMISCYYEDHQCE	71,5878	ENSG00000106633
P10153	0,105875	QHINMTSQQCTNAMQVINNYQ	71,54	ENSG00000169385
P12724	0,07330481	TIAMRAINNYRWRCKNQNTFL	71,54	ENSG00000169397
Q14435	0,05442499	SISKEYFEYIGSYDEEMEIWG	71,5082	ENSG00000115339
C9J2C3	0,05442499	SISKEYFEYIGSYDEEMEIWG	71,5082	ENSG00000115339
E7EUL0	0,05442499	SISKEYFEYIGSYDEEMEIWG	71,5082	ENSG00000115339
O60941	0,09797935	CQQCHNYQLCQNCFWRGHAGG	71,3967	ENSG00000138101
E9PEY4	0,09797935	CQQCHNYQLCQNCFWRGHAGG	71,3967	ENSG00000138101
E7EVB6	0,09797935	CQQCHNYQLCQNCFWRGHAGG	71,3967	ENSG00000138101
R4GN71	0,05882989	IQSWFRGCQVRAYIRHLNRIV	71,3648	ENSG00000162814
Q8TE57	0,11287522	GARSIRIYEMNVSTSYISVRN	71,333	ENSG00000145536
Q09MP3	0,07179926	QQVVNVENWAHYNSSTVKAHG	71,2533	ENSG00000214842
H0YEG2	0,05339526	SATYGEHIWFETNVSGDFCYV	71,2374	ENSG00000149091
Q15678	0,05657175	FHGNEEALYCNSHNSLDLNYL	71,2215	ENSG00000152104

O60674	0,06695583	HVFHIDESTRHNVLYRIRFYF	71,1896	ENSG00000096968
A0A1B0GTR9	0,06695583	HVFHIDESTRHNVLYRIRFYF	71,1896	ENSG00000096968
Q9ULJ6	0,05014214	EQFNGQNNTFSGSSYSNYSQG	71,1419	ENSG00000108175
F6WR09	0,05014214	EQFNGQNNTFSGSSYSNYSQG	71,1419	ENSG00000108175
P29323	0,06351217	MNTIRTYQVCNVFESSQNNWL	71,11	ENSG00000133216
Q6NVW1	0,06351217	MNTIRTYQVCNVFESSQNNWL	71,11	ENSG00000133216
B1AKC9	0,06351217	MNTIRTYQVCNVFESSQNNWL	71,11	ENSG00000133216
Q6ZRU5	0,06218373	GSSAWWVAVCKQVCTRVGTYA	71,0304	#N/A
Q12906	0,0917831	YGSYGYGGNSATAGYSQFYSN	71,0144	ENSG00000129351
V9GYX2	0,06612757	RVEYQCQSYYELQGSNYVTCS	71,0144	ENSG00000134365
Q92496	0,06551282	RVEYQCQSYYELQGSNYVTCS	71,0144	ENSG00000134365
A0A0C4DH21	0,06551282	RVEYQCQSYYELQGSNYVTCS	71,0144	ENSG00000134365
H7BZF3	0,06000693	KARYYLYNLMFQTWKTYVRQQ	70,9985	ENSG00000198089
Q8WYK1	0,07709834	YEQSCEVYRHQGNTAGFFYID	70,9507	ENSG00000155052
H7BXM1	0,06487566	QDLGMFLVTISCYTRGGRIIS	70,8711	ENSG00000168000
Q8N6M8	0,0852858	ARMWRIRRRYCQVLNAVRIIQ	70,8233	ENSG00000173389
Q8IZL2	0,0902506	QQQQQSSISAQQQQQQQSSIS	70,7915	ENSG00000184384
A0A087X0G5	0,0902506	QQQQQSSISAQQQQQQQSSIS	70,7915	ENSG00000184384
Q9HCJ0	0,06707012	MTMLNQLYQLQLAYQRLQIQQ	70,7915	ENSG00000078687
A0A1B0GU24	0,06707012	MTMLNQLYQLQLAYQRLQIQQ	70,7915	ENSG00000078687
Q5T4I8	0,0556674	RYGNWYARQHGSYLLSGYSYG	70,7596	ENSG00000137434
Q9H4W6	0,05422166	SSQLAVNVSETSQANDQVGYS	70,7278	ENSG00000108001
Q07507	0,07795041	VAGFQSRYFESVLDREWQFYC	70,6959	ENSG00000143196
Q15437	0,06101058	QFVTHYQHSSTQRRIRVTTIA	70,6959	ENSG00000101310
Q86TS7	0,1051753	MVQECCSQSLYYEELHSYHIV	70,68	#N/A
Q9H0C5	0,07948308	SDRIRFTVNRRISIVGFGLYG	70,6641	ENSG00000064726
A0A0U1RQI7	0,09235625	GNQALYGGQMMTSTGNQTLYW	70,6163	ENSG00000283039
C9JCQ3	0,10682809	QQQQQFQAQQSAMQQQFQAVV	70,5844	ENSG00000099917
Q96RN5	0,09282619	QQQQQFQAQQSAMQQQFQAVV	70,5844	ENSG00000099917
G3V1P5	0,09282619	QQQQQFQAQQSAMQQQFQAVV	70,5844	ENSG00000099917
H7C308	0,09282619	QQQQQFQAQQSAMQQQFQAVV	70,5844	ENSG00000099917
Q9P267	0,05474238	MSSINNTLSNHQLTHLQSLLN	70,5526	ENSG00000204406
E9PHH0	0,05474238	MSSINNTLSNHQLTHLQSLLN	70,5526	ENSG00000204406
A0A0D9SF16	0,05474238	MSSINNTLSNHQLTHLQSLLN	70,5526	ENSG00000204406
A0A1B0GW10	0,05474238	MSSINNTLSNHQLTHLQSLLN	70,5526	ENSG00000204406
A0A0D9SG23	0,05474238	MSSINNTLSNHQLTHLQSLLN	70,5526	ENSG00000204406
P05813	0,06088029	GWFNNEVGSMKIQSGAWVCYQ	70,473	ENSG00000108255
P0C7M6	0,10372894	LVQRRIRQRRQALLRVYVIQE	70,4411	ENSG00000229972
F8VUB4	0,07996259	FGMNRNQAFGMNNSLSSNIFN	70,4252	ENSG00000111596
F8VQD8	0,07996259	FGMNRNQAFGMNNSLSSNIFN	70,4252	ENSG00000111596
Q15596	0,12863835	HFGQQANTSMYSNNMNINVSM	70,3933	ENSG00000140396
H0YBB6	0,12863835	HFGQQANTSMYSNNMNINVSM	70,3933	ENSG00000140396
Q8NFP9	0,0969324	EIRCYVNGQLVSYGDMAWHVN	70,3455	ENSG00000172915
Q5T321	0,0969324	EIRCYVNGQLVSYGDMAWHVN	70,3455	ENSG00000172915
F5GXV7	0,0969324	EIRCYVNGQLVSYGDMAWHVN	70,3455	ENSG00000172915
A0A0D9SF28	0,0969324	EIRCYVNGQLVSYGDMAWHVN	70,3455	ENSG00000172915
Q9HCT0	0,07789426	VHVGVVVIKAVSSGFYVAMNR	70,3137	ENSG00000070388
Q92968	0,10647873	AYSSFSSGYGAYGNSFYGGYS	70,2818	ENSG00000162928
Q9HAK2	0,10014065	GSQLGVSISESTQGNNQGYIR	70,2659	ENSG00000221818
B7Z934	0,10014065	GSQLGVSISESTQGNNQGYIR	70,2659	ENSG00000221818
H0Y7Z8	0,08254524	STGVLGRGLDLISVRLVVNFD	70,2341	ENSG00000118197
K7ELV3	0,08316219	GAGYGSYGYGGNSATAGYNYS	70,1863	ENSG00000129351
Q01085	0,10977826	MTKNFQQVDYSQWGQWSQVYG	70,1544	ENSG00000151923
Q9NUM4	0,1158373	SIKVHNIVLMMQVTVTTTYFG	70,1226	ENSG00000106460
Q969I3	0,05209692	RFVGQFGFFEASCEWHQWTCY	70,0748	ENSG00000166840
P31942	0,06838781	GLGGYGRGGGGSGGYYGQGGM	70,0589	ENSG00000096746
Q6P3S6	0,05580397	TQRFSHSACYYDANQSMYVFG	70,0589	ENSG00000037637
P55317	0,08241898	MEGHETSDWNSYYADTQEAYS	69,9155	ENSG00000129514
C9JLN7	0,06650375	QQQQQQQQFQAQQSAMQQQFQ	69,9155	ENSG00000099917
Q3L8U1	0,0870187	MTSCSVSNSQQFSSHYSFSSN	69,8678	ENSG00000177200
H3BSP3	0,0870187	MTSCSVSNSQQFSSHYSFSSN	69,8678	ENSG00000177200
P40426	0,0533757	RHVINQTGGYSDGLGGNSLYS	69,8359	ENSG00000167081
Q5JS98	0,0533757	RHVINQTGGYSDGLGGNSLYS	69,8359	ENSG00000167081
Q92804	0,12636454	ASQSYSGYGQTTDSSYGQNYS	69,82	ENSG00000270647;ENSG00000276833

Q495T6	0,05182705	QYGNYSWDLADEQNVNGFNTL	69,82	ENSG00000142606;ENSG00 000277131
P55795	0,06941147	SMSGYDQVLQENSSDYQSNLA	69,8041	ENSG00000126945
B7ZKW0	0,05699281	HYVISLNGFLQGYNDSLQEEM	69,7563	ENSG00000101892
Q01432	0,05010183	MYANIMVLNNLRRERGLSTFL	69,6448	ENSG00000133805
E9PKC5	0,05010183	MYANIMVLNNLRRERGLSTFL	69,6448	ENSG00000133805
A0A087WXN3	0,06733346	SDRIRFSVNKRIFVVGFGLYG	69,5492	ENSG00000133243
Q9BX70	0,06502122	SDRIRFSVNKRIFVVGFGLYG	69,5492	ENSG00000133243
Q9BYJ4	0,05452387	VDVSKKTAWILGVYCRTYSRH	69,5333	ENSG00000258659
B2RNG4	0,05452387	VDVSKKTAWILGVYCRTYSRH	69,5333	ENSG00000258588
P04746	0,07041337	YDNGSNQVAFGRGNRGFIVFN	69,4378	ENSG00000243480
Q9Y485	0,06907937	RFNYQGNKFGIVDADGYLSLY	69,39	ENSG00000172869
F5H269	0,06907937	RFNYQGNKFGIVDADGYLSLY	69,39	ENSG00000172869
J3QKV3	0,07461496	RVQNEGSWNSYVDYKIFLHME	69,3741	ENSG00000002919
Q7Z353	0,06989489	LRSVFTVEQQRILQRYYENGM	69,3741	ENSG00000165259
Q9NSY1	0,07259401	QHATQQQQMLQQQFLMHSVYQ	69,3581	ENSG00000138756
H0Y9P1	0,07259401	QHATQQQQMLQQQFLMHSVYQ	69,3581	ENSG00000138756
Q2KHR3	0,07810524	SQVLSVVSLSESYASGESLTL	69,3263	ENSG00000060749
Q9NUQ7	0,07140235	DDNGWGCAYRSLQTICSWFKH	69,3103	ENSG00000109775
D6RGX2	0,07140235	DDNGWGCAYRSLQTICSWFKH	69,3103	ENSG00000109775
Q9BY15	0,06976103	YSVYCGFNAVCYNVEGSFYCQ	69,2148	ENSG00000131355
P29074	0,05938454	SYGCYQVTCHSEEGNTAYIFR	68,96	ENSG00000088179
J3KQD3	0,05938454	SYGCYQVTCHSEEGNTAYIFR	68,96	ENSG00000088179
Q9NQV6	0,07371252	GQALQQQQQQQQNSSVQHTYL	68,8963	ENSG00000170325
E9PLV1	0,07371252	GQALQQQQQQQQNSSVQHTYL	68,8963	ENSG00000170325
E9PRS0	0,07371252	GQALQQQQQQQQNSSVQHTYL	68,8963	ENSG00000170325
Q76N89	0,0509086	SSSCYSTSCYSSSCYSASCYS	68,7689	ENSG00000002746
Q8NCA5	0,06147178	QGGQFEQHFQHGGYQYNHSGF	68,7529	ENSG00000119812
Q8WYB5	0,14643458	AYNVNSVNMNMNTLNAMNGYS	68,6255	ENSG00000156650;ENSG00 000281813
Q6ZMW2	0,06456858	TFQASVSFQDVTVEFSQEEWQ	68,5937	ENSG00000196597
C9J9Y8	0,06456858	TFQASVSFQDVTVEFSQEEWQ	68,5937	ENSG00000196597
P05787	0,06223608	YGGASGMGGITAVTVNQSLLS	68,3866	ENSG00000170421
F8VUG2	0,06223608	YGGASGMGGITAVTVNQSLLS	68,3866	ENSG00000170421
F8VQY3	0,05831083	YGGASGMGGITAVTVNQSLLS	68,3866	ENSG00000170421
F8W1U3	0,05831083	YGGASGMGGITAVTVNQSLLS	68,3866	ENSG00000170421
Q92794	0,12711816	AYNVNSMNMNTLNAMNSYRMT	68,3548	ENSG00000083168
Q9Y4J8	0,08418553	QCHNYQLCQDCFWRGHAGGSH	68,2114	ENSG00000134769
Q13946	0,05266863	EIEVSVSARNIRRLLSFQRYL	68,2114	ENSG00000205268
M0QYH6	0,05018897	KDLETLKSLCRIMDNGFGNFV	68,1796	ENSG00000104960
O94916	0,09937369	MSLQSGNFLQQSSHSQAQLFH	68,1637	ENSG00000102908
P40189	0,05250454	NESSQNTSSTVQYSTVVHSGY	68,1159	ENSG00000134352
Q09327	0,05199978	MLQAVYGLDGIRLRRRQYYTM	68,1	ENSG00000128268
A0A0U1RQR8	0,06695533	QQLQALLQQQQAVMLQQLLQQ	67,9885	ENSG00000128573
A0A087WZF3	0,05417407	QSQGYNQWQQGSVHVNVLCGR	67,877	ENSG00000153187
P31483	0,13060778	AYGMYGQAWNQQGFNQTQSSA	67,8133	ENSG00000116001
F8W8I6	0,12931171	AYGMYGQAWNQQGFNQTQSSA	67,8133	ENSG00000116001
Q9UMD9	0,10255704	GQEIQQYISEYMQSDSIRSYL	67,8133	ENSG00000065618
C9JWK5	0,06584692	SAQLQLQQVALQQQQQQQQFQ	67,7974	ENSG00000099917
Q96PZ7	0,07132365	SDHSQNRQGFKLAYQAYELQN	67,7337	#N/A
F8W9C3	0,07132365	SDHSQNRQGFKLAYQAYELQN	67,7337	ENSG00000183117
E5RIG2	0,07132365	SDHSQNRQGFKLAYQAYELQN	67,7337	ENSG00000183117
F5GZ18	0,07132365	SDHSQNRQGFKLAYQAYELQN	67,7337	ENSG00000183117
A0A0U1RQY1	0,07132365	SDHSQNRQGFKLAYQAYELQN	67,7337	ENSG00000183117
H7BXU2	0,07132365	SDHSQNRQGFKLAYQAYELQN	67,7337	ENSG00000183117
O15405	0,11558891	QLQQHQMHQQIQQQMQQQHFQ	67,5107	ENSG00000103460
Q9Y2C2	0,05414448	RFLSNYFFRRFGDWRGEQNHM	67,5107	ENSG00000111962
Q86YT9	0,07782091	AKEEIVFRYYHKLRMSVEYSQ	67,4948	ENSG00000160593
E9PKK2	0,07782091	AKEEIVFRYYHKLRMSVEYSQ	67,4948	ENSG00000160593
C9JJ12	0,05601152	QTQLQLQQVALQQQQQQQQFQ	67,4948	ENSG00000099917
A0A087X1A2	0,1757121	YYGGLGYGCGGFGGLGYGYSC	67,3355	ENSG00000244362
P41221	0,05024537	GSRETAFTYAVSAAGVVNAMS	67,0807	ENSG00000114251
C9J8I8	0,05024537	GSRETAFTYAVSAAGVVNAMS	67,0807	ENSG00000114251
O00755	0,05094925	NTHQYARVWQCNCKFHWCCYV	67,0488	ENSG00000154764
Q9UGU0	0,08762422	QYEGHNVGSNAQAYGTQSNYS	67,0329	ENSG00000100207;ENSG00

				000262024;ENSG00000276461;ENSG00000280467;ENSG00000281897;ENSG00000282892;ENSG00000283026;ENSG00000283681
O15523	0,11198352	GGGGYGNSRGFGGGGYGGFYN	66,9533	ENSG00000067048
O75570	0,05585811	RLQVFRQNRNCILHLLSKNWS	66,9214	ENSG00000120662
X6RFD4	0,05585811	RLQVFRQNRNCILHLLSKNWS	66,9214	ENSG00000120662
A0A087X1S1	0,05585811	RLQVFRQNRNCILHLLSKNWS	66,9214	ENSG00000120662
Q8N6Z2	0,05585811	RLQVFRQNRNCILHLLSKNWS	66,9214	ENSG00000120662
Q8WXH2	0,05730998	LQDGYGTETYSDGGTYQGQWV	66,8737	ENSG00000154118
X6RCC3	0,05349061	HRNLGVHISRVKSVNLDQWTQ	66,8418	ENSG00000084070
Q15714	0,05430174	ERESTSGSSVSSSVSTLSHYT	66,81	ENSG00000102804
Q9NZC4	0,05021764	TDSYSTCNVSSGFFGGQWHEI	66,7781	ENSG00000135373
E9PQX0	0,05021764	TDSYSTCNVSSGFFGGQWHEI	66,7781	ENSG00000135373
E9PN75	0,05021764	TDSYSTCNVSSGFFGGQWHEI	66,7781	ENSG00000135373
E9PQR6	0,05021764	TDSYSTCNVSSGFFGGQWHEI	66,7781	ENSG00000135373
E9PPS9	0,05021764	TDSYSTCNVSSGFFGGQWHEI	66,7781	ENSG00000135373
Q00839	0,05540415	NRGYKNQSQGYNQWQQGQFWG	66,6507	ENSG00000153187
P57052	0,05216021	SFESCVKINSHNYRNEEMLVG	66,6507	ENSG00000185272
Q93074	0,07707357	QQQQQQQQQYHIRQQQQQQIL	66,587	ENSG00000184634
Q7Z3Z5	0,07707357	QQQQQQQQQYHIRQQQQQQIL	66,587	ENSG00000184634
Q13342	0,05152808	QQGQMASGDSNLNFRMVAEIQ	66,587	ENSG00000079263
P07196	0,06787228	VHISSVRSGYSTARSAYSSYS	66,5711	ENSG00000277586
A0A087X0W2	0,06787228	VHISSVRSGYSTARSAYSSYS	66,5711	ENSG00000277586
O75038	0,05905937	QMVALNYQSEGRMLQLNRAKF	66,5711	ENSG00000149527;ENSG00000276429
D6RF34	0,05124648	CRLNEYNQLQLQAAHAQEQIR	66,5711	ENSG00000126545
E9PDQ1	0,05124648	CRLNEYNQLQLQAAHAQEQIR	66,5711	ENSG00000126545
Q9HBZ2	0,0675228	TGQNMSQISRQLNQSQVAWTG	66,5392	ENSG00000172379
A0A087WVE9	0,0675228	TGQNMSQISRQLNQSQVAWTG	66,5392	ENSG00000172379
Q9H4Z2	0,06116659	TVQHLVTSDNQVQYIISQDGV	66,4596	ENSG00000198026
P46100	0,06900578	RLQQQYNQQQQQQMTYQQATL	66,4437	ENSG00000085224
Q9BUJ2	0,1338356	SYNQYQQYAQQWNQYYQNQGQ	66,4118	ENSG00000105323
B7Z4B8	0,1338356	SYNQYQQYAQQWNQYYQNQGQ	66,4118	ENSG00000105323
A0A0A0MRA5	0,1338356	SYNQYQQYAQQWNQYYQNQGQ	66,4118	ENSG00000105323
P35637	0,10053937	SGYSQSTDTSGYGQSSYSSYG	66,4118	ENSG00000089280
H3BNZ4	0,10053937	SGYSQSTDTSGYGQSSYSSYG	66,4118	ENSG00000089280
H3BPE7	0,10053937	SGYSQSTDTSGYGQSSYSSYG	66,4118	ENSG00000089280
Q9P2D1	0,05800311	SNLNQGLVNNTGMNQNLGLTN	66,4118	ENSG00000171316
P11844	0,0576794	LRVYFSRCNSIRVDSGCWMLY	66,0774	ENSG00000168582
Q7Z794	0,09137154	ISVQNSQVSVNGGAGGGGSYG	65,9977	ENSG00000189182
P04264	0,09085132	RSGYRSGGGFSSGSAGIINYQ	65,727	ENSG00000167768
H7C557	0,0613007	WCVGHERRTVYYMGYRQVYTT	65,6633	ENSG00000162591
Q5T6S3	0,05092728	MLQCYRCRQWFHEACTQCLNE	65,6473	ENSG00000119403
A0A087X169	0,05092728	MLQCYRCRQWFHEACTQCLNE	65,6473	ENSG00000119403
G3V5F8	0,08867231	QCQVCTCVVHKRCHHLIVTAC	65,5677	ENSG00000027075
G3V5U5	0,08867231	QCQVCTCVVHKRCHHLIVTAC	65,5677	ENSG00000027075
G3V304	0,08867231	QCQVCTCVVHKRCHHLIVTAC	65,5677	ENSG00000027075
G3V4Q9	0,08867231	QCQVCTCVVHKRCHHLIVTAC	65,5677	ENSG00000027075
G3V4Q6	0,08867231	QCQVCTCVVHKRCHHLIVTAC	65,5677	ENSG00000027075
G3V4X4	0,08867231	QCQVCTCVVHKRCHHLIVTAC	65,5677	ENSG00000027075
G3V3E7	0,08867231	QCQVCTCVVHKRCHHLIVTAC	65,5677	ENSG00000027075
P24723	0,08063336	QCQVCTCVVHKRCHHLIVTAC	65,5677	ENSG00000027075
O15014	0,05613261	DQSYHTHLLSTNTAYRQQYEE	65,5518	ENSG00000180357
O00571	0,09440444	FYNSDGYGGNYNSQGVDWWGN	65,4722	ENSG00000215301
A0A0C4DH59	0,05406683	GQQVTLRCSSQSGHNTVSWYQ	65,3925	ENSG00000230099
Q9Y2E5	0,09375745	NREAVLRTSTNLNSQQVIYSD	65,3288	ENSG00000013288
E9PCD7	0,09375745	NREAVLRTSTNLNSQQVIYSD	65,3288	ENSG00000013288
H0YA68	0,09375745	NREAVLRTSTNLNSQQVIYSD	65,3288	ENSG00000013288
Q9HBW9	0,05834635	GTVCIENVNANCHLDNVCIAA	65,297	ENSG00000162618
P51991	0,05665309	DGYNEGGNFGGGNYGGGGNYN	65,281	ENSG00000170144
Q92841	0,05329358	AYGTSSYTAQEYGAGTYGASS	65,1377	ENSG00000100201
H3BLZ8	0,05329358	AYGTSSYTAQEYGAGTYGASS	65,1377	ENSG00000100201
P12035	0,10457784	YGVSGGGFSSASNRGGSIKFS	65,0262	ENSG00000186442

Table S2. Selected human PrLD amyloid cores predicted aggregation propensity. AGGRESCAN [4], TANGO [5] and Zyggregator [6] predictions for the human PrLD amyloid cores candidates. Aggregation propensities values are shown for each peptide according to the corresponding predictors using the default setting and values above the aggregation threshold shown in bold.

PROTEIN	*PrLD AMYLOID CORES*	*AGGRESCAN*	*TANGO*	*ZYGGREGATOR*
**DDX5**	TQNGVYSAANYTNGSFGSNFV	-10.6	1.28	-3.66
**EYA1**	MQGSSFTTSSGIYTGNNSLTN	-12.3	0	-3.65
**ILF3**	YGSYGYGGNSATAGYSQFYSN	-3.1	0	-3.72
**MED15**	QQQQQFQAQQSAMQQQFQAVV	-44.6	0	-4.63
**NCOA2**	HFGQQANTSMYSNNMNINVSM	-20.9	0	-1.17
**PHC1**	QQQQIHLQQKQVVIQQQIAIH	-17.5	**57.17**	-2.10
**TIA1**	AYGMYGQAWNQQGFNQTQSSA	-30.9	0	-4.09

Table S3. Selected human PrLD amyloid cores hydrophaticity. For each candidate, the grand average of hydropathy **(**GRAVY) value was evaluated using the EXPASY ProtParam tool [7]. Positive values corresponding to hydrophobic sequences are shown in bold. α-synuclein (ASYN) and Aβ42 short amyloidogenic stretches were predicted with AmylPred2 [8].

PROTEIN	***PrLD AMYLOID CORES***	***GRAVY SCORE***
**DDX5**	TQNGVYSAANYTNGSFGSNFV	-0.357
**EYA1**	MQGSSFTTSSGIYTGNNSLTN	-0.49
**ILF3**	YGSYGYGGNSATAGYSQFYSN	-0.786
**MED15**	QQQQQFQAQQSAMQQQFQAVV	-1.024
**NCOA2**	HFGQQANTSMYSNNMNINVSM	-0.643
**PHC1**	QQQQIHLQQKQVVIQQQIAIH	-0.633
**TIA1**	AYGMYGQAWNQQGFNQTQSSA	-1.019
**ASYN**	GVLYVG	**1.683**
	GGAVVTGVTAVAQ	**1.238**
**Aβ42**	GAIIGLMVGGVVI	**2.462**
	QKLVFFAE	**0.562**

Table S4. Disorder context of PrLD amyloid cores. FoldIndex [9], IUPRED [10], PONDR-FIT [11] and RONN [12] algorithms were used for disorder prediction. Disorder was analyzed for the 21 residues-long peptides and 20 flanking residues at each end and expressed as the percentage of disordered residues in these 61 residues-long segments. Average disorder accounts for the mean of all disorder predictions for a given segment.

DISORDER PREDICTOR	***DDX5***	***EYA1***	***ILF3***	***MED15***	***NCOA2***	***PHC1***	***TIA1***
**FOLDINDEX**	91	61	74	100	25	62	100
**IUPRED**	37	75	64	31	100	69	38
**PONDR-FIT**	51	100	100	100	100	100	56
**RONN**	41	100	100	100	100	85	51
**AVERAGE**	**55**	**84**	**85**	**83**	**82**	**79**	**61**

Table S5. **Selected human PrLD amyloid cores secondary structure assignment**. Assignment and area of the secondary structure components of aggregated PrLD amyloid cores in the amide I region of the FTIR spectra.

ASSIGNMENTS	***DDX5***	***EYA1***	***ILF3***	***MED15***	***NCOA2***	***PHC1***	***TIA1***
**Inter β-sheet (1623-1641 cm^{-1})**	37%	67.5%	46%	54%	52%	66.5%	62%
**Disordered/Loops/Turns (1658-1665 cm^{-1})**	63%	32.5%	34%	35%	24%	33.5%	31.5%
**β-sheet (1674-1695 cm^{-1})**	-	-	20%	11%	24%	-	6.5%

Table S6. Protein-protein interactions (PPI) statistics for human PrLD-containing proteins. The PPI enrichment p-value was obtained from the STRING database [13] and reflects the relationship between the number of interactions established by the different proteins and those randomly expected.

PROTEIN	***PPI enrichment p-value***
**DDX5**	7.76e-06
**EYA1**	1.12e-10
**ILF3**	0.00294
**MED15**	1.87e-10
**NCOA2**	2.03e-12
**PHC1**	0
**TIA1**	0.45

References:

1 Toombs, J. a. *et al.* De novo design of synthetic prion domains. *Proceedings of the National Academy of Sciences* **109**, 6519-6524, (2012).
2 Sabate, R., Rousseau, F., Schymkowitz, J. & Ventura, S. What Makes a Protein Sequence a Prion? *PLoS Computational Biology* **11**, e1004013, (2015).
3 UniProt Consortium, T. U. UniProt: a hub for protein information. *Nucleic acids research* **43**, D204-212, (2015).
4 Conchillo-Sole, O. *et al.* AGGRESCAN: a server for the prediction and evaluation of "hot spots" of aggregation in polypeptides. *BMC bioinformatics* **8**, 65, (2007).
5 Fernandez-Escamilla, A.-M., Rousseau, F., Schymkowitz, J. & Serrano, L. Prediction of sequence-dependent and mutational effects on the aggregation of peptides and proteins. *Nature biotechnology* **22**, 1302-1306, (2004).
6 Tartaglia, G. G. & Vendruscolo, M. The Zyggregator method for predicting protein aggregation propensities. *Chemical Society reviews* **37**, 1395-1401, (2008).
7 Artimo, P. *et al.* ExPASy: SIB bioinformatics resource portal. *Nucleic Acids Res* **40**, W597-603, (2012).
8 Tsolis, A. C., Papandreou, N. C., Iconomidou, V. A. & Hamodrakas, S. J. A consensus method for the prediction of 'aggregation-prone' peptides in globular proteins. *PloS one* **8**, e54175, (2013).
9 Prilusky, J. *et al.* FoldIndex©: A simple tool to predict whether a given protein sequence is intrinsically unfolded. *Bioinformatics* **21**, 3435-3438, (2005).
10 Dosztanyi, Z., Csizmok, V., Tompa, P. & Simon, I. IUPred: web server for the prediction of intrinsically unstructured regions of proteins based on estimated energy content. *Bioinformatics* **21**, 3433-3434, (2005).
11 Xue, B., Dunbrack, R. L., Williams, R. W., Dunker, A. K. & Uversky, V. N. PONDR-FIT: a meta-predictor of intrinsically disordered amino acids. *Biochimica et biophysica acta* **1804**, 996-1010, (2010).
12 Yang, Z. R., Thomson, R., McNeil, P. & Esnouf, R. M. RONN: the bio-basis function neural network technique applied to the detection of natively disordered regions in proteins. *Bioinformatics* **21**, 3369-3376, (2005).
13 Szklarczyk, D. *et al.* STRING v10: protein-protein interaction networks, integrated over the tree of life. *Nucleic Acids Res* **43**, D447-452, (2015).
